# Design of a Multi-Epitopes Vaccine against Hantaviruses: An Immunoinformatics and Molecular Modelling Approach

**DOI:** 10.3390/vaccines10030378

**Published:** 2022-02-28

**Authors:** Saba Ismail, Sumra Wajid Abbasi, Maha Yousaf, Sajjad Ahmad, Khalid Muhammad, Yasir Waheed

**Affiliations:** 1 Foundation University Medical College, Foundation University Islamabad, Islamabad 44000, Pakistan; saba.ismail@fui.edu.pk; 2 NUMS Department of Biological Sciences, National University of Medical Sciences, Abid Majeed Rd, The Mall, Rawalpindi 46000, Pakistan; sumra.abbasi@numspak.edu.pk; 3 Department of Biosciences, COMSATS University Islamabad, Islamabad 45550, Pakistan; maha.yousaf.vt8086@iiu.edu.pk; 4 Department of Health and Biological Sciences, Abasyn University, Peshawar 25000, Pakistan; sahmad@bs.qau.edu.pk; 5 Department of Biology, College of Science, United Arab Emirates University, Al Ain 15551, United Arab Emirates

**Keywords:** hantaviruses, ViPR database, multi-epitope database, molecular dynamics simulations, binding free energies

## Abstract

Hantaviruses are negative-sense, enveloped, single-stranded RNA viruses of the family Hantaviridae. In recent years, rodent-borne hantaviruses have emerged as novel zoonotic viruses posing a substantial health issue and socioeconomic burden. In the current research, a reverse vaccinology approach was applied to design a multi-epitope-based vaccine against hantavirus. A set of 340 experimentally reported epitopes were retrieved from Virus Pathogen Database and Analysis Resource (ViPR) and subjected to different analyses such as antigenicity, allergenicity, solubility, IFN gamma, toxicity, and virulent checks. Finally, 10 epitopes which cleared all the filters used were linked with each other through specific GPGPG linkers to construct a multi-antigenic epitope vaccine. The designed vaccine was then joined to three different adjuvants—TLR4-agonist adjuvant, β-defensin, and 50S ribosomal protein L7/L12—using an EAAAK linker to boost up immune-stimulating responses and check the potency of vaccine with each adjuvant. The designed vaccine structures were modelled and subjected to error refinement and disulphide engineering to enhance their stability. To understand the vaccine binding affinity with immune cell receptors, molecular docking was performed between the designed vaccines and TLR4; the docked complex with a low level of global energy was then subjected to molecular dynamics simulations to validate the docking results and dynamic behaviour. The docking binding energy of vaccines with TLR4 is −29.63 kcal/mol (TLR4-agonist), −3.41 kcal/mol (β-defensin), and −11.03 kcal/mol (50S ribosomal protein L7/L12). The systems dynamics revealed all three systems to be highly stable with a root-mean-square deviation (RMSD) value within 3 Å. To test docking predictions and determine dominant interaction energies, binding free energies of vaccine(s)–TLR4 complexes were calculated. The net binding energy of the systems was as follows: TLR4-agonist vaccine with TLR4 (MM–GBSA, −1628.47 kcal/mol and MM–PBSA, −37.75 kcal/mol); 50S ribosomal protein L7/L12 vaccine with TLR4 complex (MM–GBSA, −194.62 kcal/mol and MM–PBSA, −150.67 kcal/mol); β-defensin vaccine with TLR4 complex (MM–GBSA, −9.80 kcal/mol and MM–PBSA, −42.34 kcal/mol). Finally, these findings may aid experimental vaccinologists in developing a very potent hantavirus vaccine.

## 1. Introduction

The emergence and rise in the spread of RNA viruses in recent years have posed major threats to human life [1]. Hantaviruses are negative-sense, enveloped, single-stranded RNA viruses, hosted by small mammals such as rodents, shrews, bats, and moles [2]. They are responsible for the occurrence of a zoonotic disease named hantavirus cardiopulmonary syndrome (HCS) in America and haemorrhagic fever with renal syndrome (HFSR) in Europe. Hantaviruses infect 150,000 to 200,000 humans annually, with a case fatality rate of 0.1% to 50% based on the species with a relatively higher prevalence in Asia [3,4,5,6]. In addition to Asia, around 3000 HFRS cases are identified each year in Europe [6]. HFRS instances have also been documented in Singapore, Vietnam, Thailand, Sri Lanka, and India, as well as in Sweden, Finland, Germany, France, Switzerland, the Balkans, the Czech Republic, Poland, Greece, Lithuania, Estonia, Slovenia, Turkey, and the United Kingdom [6].

Structurally, hantaviruses are encapsulated with multiple copies of nucleoproteins and the viral RNA is composed of three segments: The largest segment is RNA polymerase, the middle size segment encodes for glycoproteins such as glycoproteins Gn, glycoproteins Gc, and the small segment produces a nucleocapsid protein [7]. Generally, hantaviruses are classified into three different classes based on their associated reservoir host. The first class is of old world viruses such as Seoul virus (SEOV), Hantaan virus (HNTV), and Dobrava–Belgrade virus (DOBV) responsible for HFRS. These are hosted by Murinae rodents predominantly found in Europe and Asia. The second class is of New World viruses such as New York-1 virus (NY-1V), Sin Nombre virus (SNV), and Andes virus (ANDV), causing hantavirus pulmonary syndrome (HPS). These viruses are transmitted via Sigmondontinae subfamily members mostly found in the US. The third class comprises both Old and New World hantaviruses such as Prospect Hill Virus (PHV), Puumala virus (PUUV), and Tula virus (TULV) harboured by Arvicolinae rodents [8]. Extensive bioinformatics and phylogenetic analysis have revealed that the progression of hantaviruses from bats, shrews, and moles to rodents is a consequence of the coevolution of viruses and host and geographic divergence [9,10,11]. Such coevolution of viruses in rodents is responsible for the emergence of virulent hantaviruses capable of infecting humans [12].

Hantavirus disease is transmitted by infected urine, faeces, and saliva, as well as by biting and aerosolised filth breathed by healthy rats [13,14]. The human kidney and lungs are considered to be the primary targets of HFRS-associated and HCPS-associated viruses, respectively [6]. Males have a threefold higher rate of infection than females [6]. Symptoms of hantavirus disease include muscle pain, high fever, gastrointestinal (GI) symptoms, and vascular leakage [15]. Hantaviruses typically infect vascular endothelial cells in humans, so they malfunction in capillaries and other vessels [16]. As a result, the core pathophysiology of hantavirus-associated illnesses is a substantial increase in vascular permeability [16]. Replication of hantavirus occurs in the vascular endothelium and macrophages specifically present in the kidney and lungs [17]. Virulent hantaviruses infect host cells by adhering to the αγβ3 integrin proteins present on the cell membrane, ultimately leading to phagocytosis [18].

The immune system plays a crucial role in the pathogenesis of hantavirus disease [6], as well as in the fight against cancers and viral infections [19]. Immunotherapy is a potent and effective technique for the prevention of infectious illnesses nowadays [20]. Multi-epitope vaccination has recently been discovered to be an incredible strategy for the prevention and treatment of cancers and viral infections [21,22,23,24,25]. An optimal multi-epitope vaccine construct should have a sequence of overlapping epitopes, with each antigenic peptide fragment eliciting either a cellular or a humoral immune response against the tumour or virus of interest [19]. Thus far, many therapeutics and vaccines against hantaviruses have been developed; these include virus-like particle (VLP)-based vaccines, recombinant proteins, virus-vectored recombinant vaccines, and nucleic acid-based molecular vaccines [3]. Hantavax is a commercialised, formalin-inactivated vaccine for HFRS, prepared against HTNV growth inside the brains of mice [3,26]. Initially, Hantavax was found to elicit a strong immune response; however, it was failed to establish a statistically significant decrease in HFRS disease intensity [27]. Similarly, CD40 ligand (CD40L) and granulocyte-macrophage colony-stimulating factor (GM-CSF) Hantaan VLPs were developed in 2019 and found to boost long-term immunity against HTNV infection substantially [28]. However, a lack of evaluation of vaccine protective efficacy meant these VLPs did not progress into clinical trials [3]. Like the above-mentioned vaccines, many Recombinant N proteins such as PUUV N, ANDV N, DOBV N [29], virus-vectored recombinant vaccines such as VACV-vectored HTNV [30], Ad-vectored ANDV [31], VSV-vectored ANDV [32], and nucleic acid-based molecular vaccines, i.e., HTNV DNA [33], PUUV DNA [33], pVAX-LAMP/Gn, and pVAX-LAMP/Gc [34] have been developed so far by experimental vaccinologists, but no vaccine or drug has yet been approved by the FDA [17].

**Figure 1:**
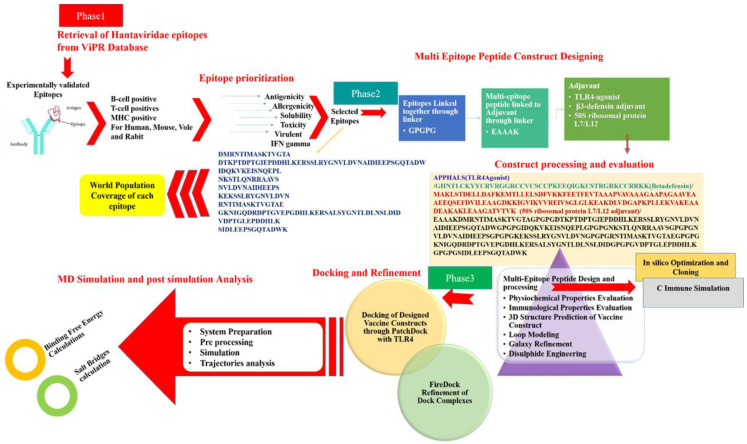
The schematic representation of steps followed throughout the study.

The research performed in this study used applications of reverse vaccinology (RV), in combination with biophysical approaches, to construct a multi-epitope vaccine and decipher its binding potential with host immune system components, as well as evaluate its potential in providing immune protection against hantaviruses. To the best of our knowledge, no such previous effort is reported where experimental immune protective epitopes are used in a computational vaccine pipeline. This study is novel, as the focus was more on experimentally proved immune protective epitopes rather than using predicted epitopes from the virus proteins. The study is also helpful in overcoming the limited antigenicity and immunogenicity of the epitopes by modeling them into a multi-epitope peptide vaccine, in which only epitopes were selected that broadly cover all successful parameters of epitope-based vaccines (Figure 1).

## 2. Materials and Methods

### 2.1. Target Epitopes Retrieval

The study started with the retrieval of experimentally determined epitopes of hantaviruses from the ViPR [35]. The Allergenicity of peptides was evaluated using the AllerTOP2.0 web tool (Department of Pharmacy, Medical University of Sofia, Sofia, Bulgaria) [36]. Non-allergen peptides were selected, and a web-server VaxiJen 2.0 [37] was used, with a threshold greater than 0.4, to reveal the antigenicity of the selected epitopes. The ToxinPred [38] tool was then used to check the toxicity. Similarly, Virulentpred [39] was used to check the virulence potential of the selected nonallergen and nontoxic peptides [39]. To examine the solubility of the peptides, the Innovagen webserver (http://www.innovagen.com/proteomics-tools (14 December 2020)) was adopted. Using an IFN epitope server, epitopes were then tested for their capacity to produce IFN-γ. Peptides showing positive IFN-γ inducer were selected and further screened for exposed peptides via TMHMM 2.0 [40]. Peptides located on the outer side of the cell membrane were considered for further scrutinisation [40].

### 2.2. Chimeric Vaccine Designing

Peptide vaccines usually are weak immunogens, which can be enhanced by fusing multiple immunodominant epitopes to create a multi-epitope peptide vaccine [41]. These multi-epitope vaccines are considered a promising preventing option for bacterial and viral infections [42,43]. A multi-epitope subunit vaccine also contains a strong immunostimulatory adjuvant for enhancing immunogenicity and activating long-lasting innate and adaptive immune responses [44]. In this study, we designed three vaccine constructs, each with different adjuvants—TLR4-agonist, β-defensin, and 50S ribosomal protein L7/L12. The linkers which were used to join the finalised epitopes were GPGPG linkers [44]. The EAAAK linker was used as a stiff spacer to bind the adjuvant’s N terminal to the epitope peptide [45].

### 2.3. Physicochemical and Immunological Properties

Physicochemical properties of the constructs were analysed using Protparam of the ExPASy server [46]. Immunological properties, such as antigenicity and allergenicity [36], were evaluated for all 3 vaccines using Vaxijen [37] and Allertop [36]. Modelling of the tertiary structure of the vaccines was performed via 3Dpro of SCRATCH protein predictor [47]. The PDBSum [48] structural database was used to analyse Ramachandran plots for the vaccines [48]. Loop modelling was performed via the GalaxyLoop tool of GalaxyWeb (http://galaxy.seoklab.org/ (24 December 2020)), and refinement of the structure was performed with GalaxyRefine [49].

### 2.4. Molecular Docking

To generate effective immune responses, it is crucial to understand the binding pattern between the designed vaccines and the TLR4 immune cell receptor [50]. TLR4 expression is reported to be upregulated and mediates the secretion of several cytokines in hantavirus infection [51]. To achieve this objective, molecular docking of vaccines and TLR4 receptor (PDB ID: 4G8A) was performed via the online webserver PatchDock (http://bioinfo3d.cs.tau.ac.il/PatchDock/ (1 December 2021)) [52]. For receptor preparation and preprocessing, all heteroatoms and cocrystallised ligands were removed from TLR4 3D structure via UCSF Chimera. Results of molecular docking were further refined via FireDock (http://bioinfo3d.cs.tau.ac.il/FireDock/ (2 January 2021)) [53,54] and visualised by Chimera 1.15 [55], PDBSum [48] and DIMPLOT [56].

### 2.5. Molecular Dynamics Simulations

Molecular dynamic simulations were performed using AMBER18 [57,58]. Initial libraries of the complexes were prepared with an Antechamber module [59]. The receptor–vaccine complexes were solvated in a TIP3P water box, and the boundary size was set at 12 Ǻ [60]. To parameterise the receptor and vaccine molecules, force fields, i.e., ‘ff14SB’ [61,62] were used, respectively. In total, 12 Na+ ions were added to neutralise the system. Hydrogen atoms were minimised for 500 cycles, water box for 1000 rounds, α-carbon atoms for 1000 cycles, and nonheavy atoms for 300 cycles, allowing a restraint of 200 kcal/mol Å^2^, 5 kcal/mol Å^2^, and 100 kcal/mol Å^2^ on the rest of system, respectively [50]. After that, the systems were heated at 300 K for 20 ps in an NVT ensemble under periodic boundary conditions, and the hydrogen bond atoms were restrained by applying the SHAKE algorithm [63]. System equilibration was performed for 100 ps. Along with this, pressure equilibration was performed in the NPT ensemble first with restraint on α-carbon atoms and the second without restraint for 50 ps. After completing the above-mentioned steps, system equilibration was accomplished. A distance of 0.8 Å was set as the default cutoff value for nonbonded interactions, and the production run was executed for 100 ns. CPPTRAJ [64] command was utilised to determine trajectories. Binding free energies of the complexes were calculated via MMPB/GBSA method implemented in AMBER18 [58]. The following equation was used to estimate the systems net binding energy:

ΔGbind = ΔGcomplex − [ΔGreceptor + ΔGligand].(1)

In total, 500 frames were picked from MD trajectories, each after every 0.2 ns.

### 2.6. Computational Immune Simulation

The immunogenic potential of the vaccine was evaluated by performing computational immune simulations via the C-ImmSim [65] server, which uses a position-specific score matrix (PSSM) and various other machine learning techniques to predict and study epitope and immune interactions. The immune simulation studies of the vaccines were performed using the following parameters: dose gap of two to four weeks; time steps of the 3 injections were set at 1, 84, and 168 over 4 weeks.

### 2.7. Disulphide Engineering and Codon Optimisation

To enhance the structural stability of the vaccines, disulphide bonds were created in the target modelled vaccine. Disulphide engineering is a recent and novel approach and can be performed with the help of Disulphide by Design 2.12 webserver [66]. This webserver also yields those pairs of amino acids which can be considered as the final target for disulphide engineering. To obtain a higher expression of the cloned sequence in the expression system (*Escherichia coli*), the sequence of the vaccine construct was reverse translated and optimised for codon usage via Java Codon Adaptation Tool (JCat) [67]. Codon adaptation index (CAI) and the percentage of GC were used to evaluate the expression of the cloned sequences. Ideally, the value of CAI should be 1, and the GC content of the refined sequence should be between 30–70%, which depicts favourable transcriptional and translational efficiencies [68,69,70]. Some additional input factors were also considered to prevent rho-independent transcription termination [50]. Finally, SnapGene (https://www.snapgene.com/ (10 January 2021) was used to clone the optimised vaccine construct in the pET-28a (+) expression vector.

## 3. Results and Discussion

### 3.1. Epitopes Analysis

In this study, three multi-epitopes vaccines were designed using experimentally determined human immune system stimulating epitopes against hantaviruses. The objective was to obtain appropriate epitopes among the catalogued epitopes with suitable vaccine properties. Epitopes were screened based on several parameters such as allergenicity, antigenicity, presence of transmembrane helices, toxicity, solubility, IFN-positivity, and virulence. After all these analyses, the selected epitopes were joined with each other through GPGPG linker and joined with three different types of adjuvants: (1) TLR4-agonist, (2) β-defensin, and (3) 50S ribosomal protein L7/L12 adjuvant, with the help of EAAAK linker for boosting of immune reactions. To create a more stable vaccine design, a GPGPG linker was inserted between the epitope sequences. The linkers helped in the functional preservation of each epitope (9–15 residues) so that they could act independently after being imported into the human body. Previous research has shown that utilising EAAAK as a linker increases the bioactivity of the vaccine fusion protein; thus, it was placed in the N terminal of the fusion peptide [71]. For designing the vaccine construct, 336 experimentally determined epitopes (Table S1) were retrieved that are experimentally validated immune protectors. Out of 336 epitopes, 140 allergens, 45 nonantigenic, 40 poorly soluble, and 26 showed negative results for IFN-production, and these were discarded. In total, among 84 IFN-positive epitopes, 74 nonvirulent epitopes were discarded, and 10 epitopes were predicted as virulent. Finally, 10 epitopes were shortlisted: DMRNTIMASKTVGTA, DTKPTDPTGIEPDDHLKERSSLRYGNVLDVNAIDIEEPSGQTADW, IDQKVKEISNQEPL, NKSTLQNRRAAVS, NVLDVNAIDIEEPS, KEKSSLRYGNVLDVN, RNTIMASKTVGTAE, GKNIGQDRDPTGVEPGDHLKERSALSYGNTLDLNSLDID, VDPTGLEPDDHLK, and SIDLEEPSGQTADWK. All selected epitopes were exposed. The antigenicity of selected epitopes ranged from 0.709 to 1.067, which was higher than the default threshold of 0.4, which is typically used for viruses. Furthermore, the virulence score was found to be between 0.99 and 1.05, as tabulated in Table 1, which was also greater than the default threshold of 0.0. These 10 filtered epitopes were then considered for the design of vaccine constructs.

### 3.2. Population Coverage Analysis

**Figure 2:**
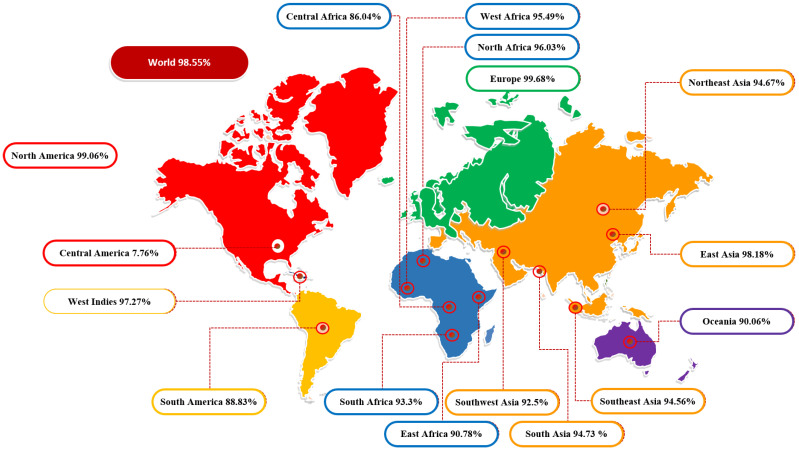
Diagrammatic representation of global population coverage of selected epitopes.

The molecules of the major histocompatibility complex (MHC) are highly polymeric and extensively dispersed across the world’s population. As a result, using a multi-epitope peptide-based vaccine to develop a broad-spectrum vaccine that does not target a single ethnic group is a rational strategy. The global population coverage was provided by the IEDB analysis tool to predict MHC I and MCH II combined population coverage using the final 10 selected epitopes shown in Figure 2. These epitopes interact with a collection of reference alleles, including HLA-A*01:01; HLA-A*02:01; HLA-A*02:01; HLA-A*02:03; HLA-A*02:03; HLA-A*02:06; HLA-A*02:06; HLA-A*03:01; HLA-A*03:01; HLA-A*11:01; HLA-A*11:01; HLA-A*23:01; HLA-B*08:01; HLA-A*23:01; HLA-A*24:02; HLA-A*24:02; HLA-A*26:01; HLA-A*26:01; HLA-A*30:01; HLA-A*30:01; HLA-B*57:01; HLA-A*30:02; HLA-A*31:01; HLA-B*58:01; HLA-A*32:01; HLA-A*33:01; HLA-A*33:01; HLA-A*68:01; HLA-A*68:01; HLA-A*68:02; HLA-A*68:02; HLA-A*30:02; HLA-B*07:02; HLA-B*51:01; HLA-B*07:02; HLA-B*08:01; HLA-B*15:01; HLA-B*15:01; HLA-B*35:01; HLA-A*31:01; HLA-B*35:01; HLA-B*40:01; HLA-B*40:01; HLA-B*44:02; HLA-B*44:02; HLA-B*44:03; HLA-B*44:03; HLA-B*51:01; HLA-A*01:01; HLA-B*53:01; HLA-B*53:01; HLA-B*57:01; HLA-A*32:01; HLA-B*58:01 for MHCI and HLA-DRB4*01:01; HLA-DRB1*04:01; HLA-DRB1*04:05; HLA-DRB1*07:01; HLA-DRB1*09:01; HLA-DRB1*11:01; HLA-DRB1*03:01; HLA-DRB1*13:02; HLA-DRB1*15:01; HLA-DRB3*01:01; HLA-DRB1*12:01; HLA-DRB3*02:02; HLA-DRB1*08:02; HLA-DRB1*01:01; HLA-DRB5*01:01 for MHCIIs. The countrywide population coverage for MHCI, MHCII and their combination is tabulated in Table S2. The results showed that the world population coverage was 98.55% for combined MHC class I and II. Furthermore, the predicted values for PC90 were 15.14 and 5.5 for MCH-I and MCH-II, respectively. In brief, our assessment confirmed that selected epitopes could be potential candidates for the development of multi-epitope vaccine constructs.

### 3.3. Vaccine with Different Adjuvants

**Figure 3:**
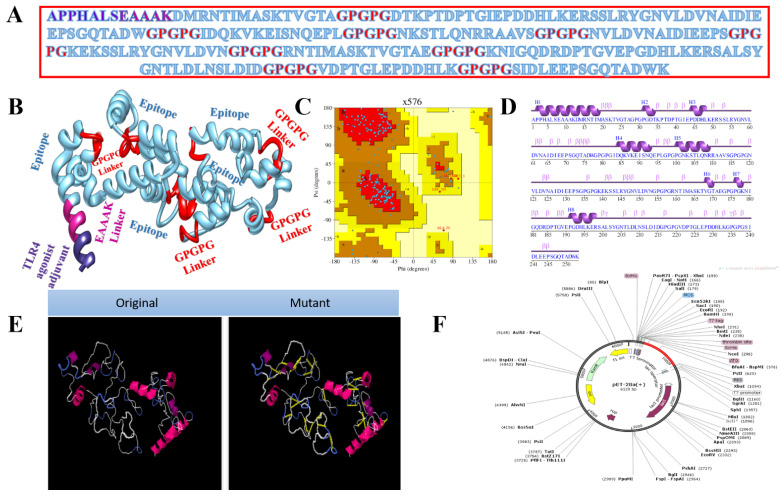
Structure characterisation of model vaccine with TLR4-agonist adjuvant (**A**) amino acid sequence, (**B**) 3D structure of TLR4-agonist vaccine, (**C**) Ramachandran plot, (**D**) secondary structure elements, (**E**) original versus mutated structure, and (**F**) cloned vaccine into expression vector.

Vaccines based on entire organisms and proteins have proven effective in decreasing mortality and morbidity caused by infectious agents. However, high antigenic load results in inaccurate immune responses and is associated with reactogenic reactions. Multi-epitopes vaccines provide an appealing option, as they can be easily synthesised, are cost-efficient, are easy to produce, and produce specific immune responses. However, peptide vaccines are weakly immunogenic. Adjuvant addition can improve the immunogenicity of peptide multi-epitope vaccines. To design a multi-epitope vaccine construct, all selected epitopes were linked to each other with a specific linker GPGPG. As an adjuvant, TLR4-agonist was joined to increase the immunogenicity of constructs through EAAK linker to enhance immune responses against the antigen. The total count of amino acid residues for the final construct was 253. The complete amino acid sequence of the vaccine is shown in Figure 3A, while the vaccine 3D model is presented in Figure 3B. The 3D model of the vaccine contains 82.9% amino acids in favoured regions, 13.8% in additional allowed regions, and 1.1% in the disallowed regions of the Ramachandran plot (Figure 3C). The secondary structure elements of the vaccine depicted 10 helices, 6 helix–helix interactions, 58 beta turns, and 2 gamma turns (Figure 3D). Similarly, the vaccine wild and mutant structures are shown in Figure 3E, while its cloned sequence is shown in Figure 3F.

**Figure 4:**
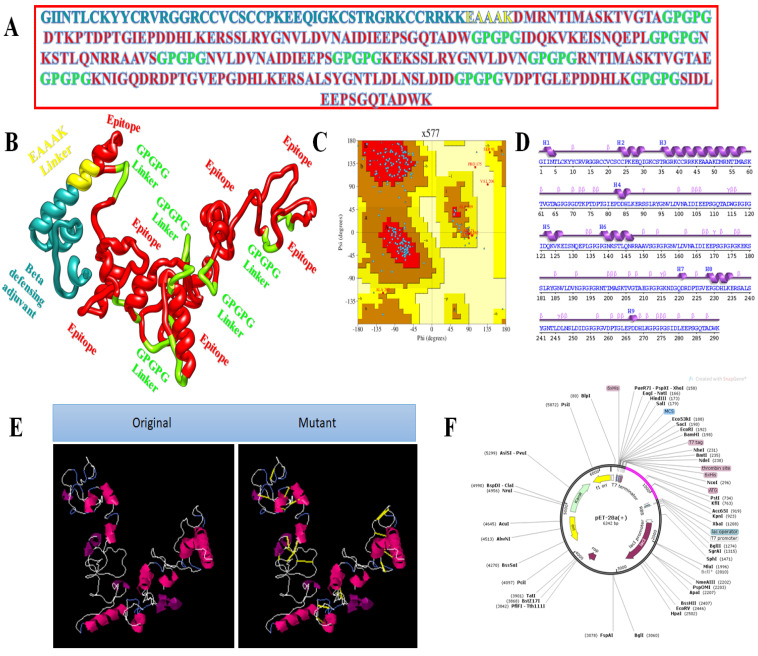
Structure characterisation of model vaccine with β-defensin adjuvant (**A**) amino acid sequence, (**B**) 3D structure of β-defensin vaccine, (**C**) Ramachandran plot, (**D**) secondary structure elements, (**E**) original versus mutated structure, and (**F**) cloned vaccine into expression vector.

The second vaccine construct has β-defensin as an adjuvant joined through the EAAAK linker. The amino acid length is 291. The complete amino acid sequence of the vaccine is presented in Figure 4A, while the vaccine 3D model is shown in Figure 4B. The 3D model of the vaccine contains 82.4% amino acids in favoured regions, 14.4% in additional allowed regions, and 0.5% in the disallowed regions of the Ramachandran plot (Figure 4C). The secondary structural elements of the vaccine depicted 14 helices, 5 helix–helix interactions, 35 beta turns, and 1 gamma turns (Figure 4D). The original versus mutated structures are presented in Figure 4E, and in silico cloning analyses are presented in Figure 4F.

**Figure 5:**
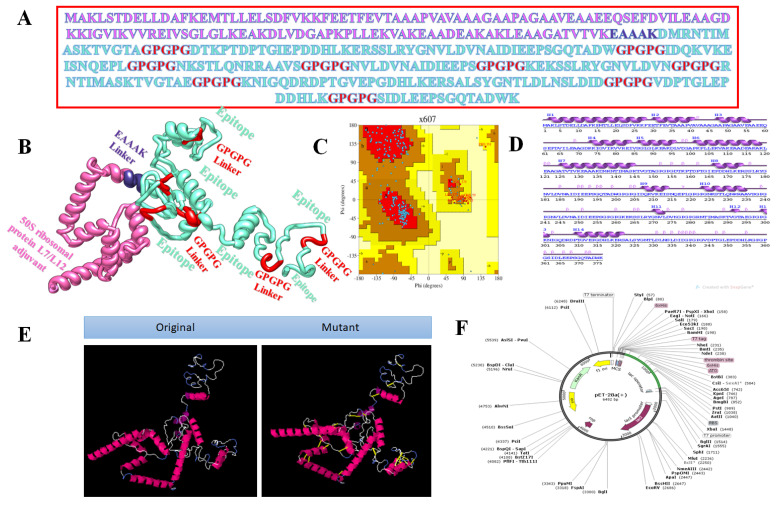
Structure characterisation of model vaccine with TLR4-agonist adjuvant (**A**) amino acid sequence, (**B**) 3D structure of 50S Ribosomal protein L7/L12 vaccine, (**C**) Ramachandran plot, (**D**) secondary structure elements, (**E**) original versus mutated structure, and (**F**) cloned vaccine into expression vector.

The third vaccine construct has 50S ribosomal protein L7/Ll2 as an adjuvant molecule. The vaccine is 376 amino acids in length. The complete amino acid sequence of the vaccine is shown in Figure 5A, while the vaccine 3D model is presented in Figure 5B. The 3D model of the vaccine contains 88.1 residues in favourable regions, 9.2% in additional allowed regions, and 0.0% in the disallowed regions of the Ramachandran plot (Figure 5C). The secondary structural elements of the vaccine depicted 15 helices, 9 helix–helix interactions, 49 beta turns, and 2 gamma turns (Figure 5D), and the original versus mutated structures are presented in Figure 5E, and in silico cloning analyses are presented in Figure 5F.

### 3.4. Multi-Epitope Vaccine Design

**Figure 6:**
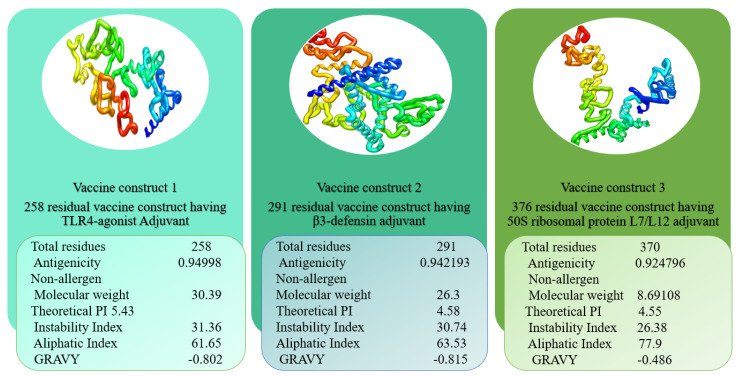
Vaccine constructs with different three types of adjuvants.

After designing the vaccines, their physicochemical characteristics were determined and are summarised in Figure 6. As can be observed, the construct is very thermally stable and easy to handle during experimental analysis due to its tiny size. The numbers of amino acids were in the range of 253–376. The TLR4-agonist vaccine and β-defensin vaccine constructs had similar GRAVY values (−0.802, −0.815), and the 50S ribosomal protein L7/L12 vaccine had a value of −0.486. The GRAVY value is an assessment of the hydrophilicity and hydrophobicity of a given sequence. The GRAVY value was negative for all predicted structures, signifying their hydrophilic nature. Their theoretical pI values were shown to be 5.43, 4.58, and 4.55 for TLR4-agonist, β-defensin, and 50S ribosomal protein L7/L12 vaccines, respectively. The instability index prediction demonstrated slight differences among these three constructs in the range of 26.38–31.36. The instability indices of TLR4-agonist, β-defensin, and 50S ribosomal protein L7/L12 vaccine constructs were 31.36, 30.74, and 26.38, respectively, which categorised the protein structures as stable (score *>* 40 directs instability). Similarly, an aliphatic index of 61.65 (TLR4-agonist), 63.53 (β-defensin), and 77.9 (50S ribosomal protein L7/L12 vaccine) indicated the thermostability of the vaccine constructs. Moreover, all these vaccines were confirmed to be nonallergenic, and their antigenicity values were calculated to be > 0.92. The overall content of amino acids of the construct structures was similar, and the only difference was that of selected adjuvants. As a result, no major alterations in physicochemical properties were observed. The construct’s stable and functioning three-dimensional structural unit was anticipated and subjected to loop modelling. Following that, the loop-modelled construct was refined. Both local and global searches were conducted with a greater degree of constraint. Table 2 illustrates the various refinement parameters for the top five most refined structures of constructs. Model 1 was chosen as the refined model because it had the lowest stable galaxy energy, a lower MolProbity and clash score, no bad rotamers, and the greatest number of Rama-favoured residues in comparison with the original input structure.

Disulphide engineering of the vaccines was performed to optimise molecular interactions and confer considerable stability by attaining precise geometric conformation. In the process, pairs of residues which had an interaction energy nonfavourable towards vaccine stability were mutated to cysteine. The binding energy value of the mutated residues was > 1 kcal/mol. For the TLR-4-agonist vaccine adjuvant, 26 pairs of residues were mutated. Likewise, in the β-defensin vaccine construct, 23 pairs of residues were replaced with cysteine amino acids. Similarly, in the 50S ribosomal protein L7/L12 vaccine construct, 26 pairs of residues were found highly unstable hence mutated. All unstable residue pairs, along with their binding energy, are tabulated in Table S3.

### 3.5. Molecular Docking of Vaccine Constructs with TLR4

**Figure 7:**
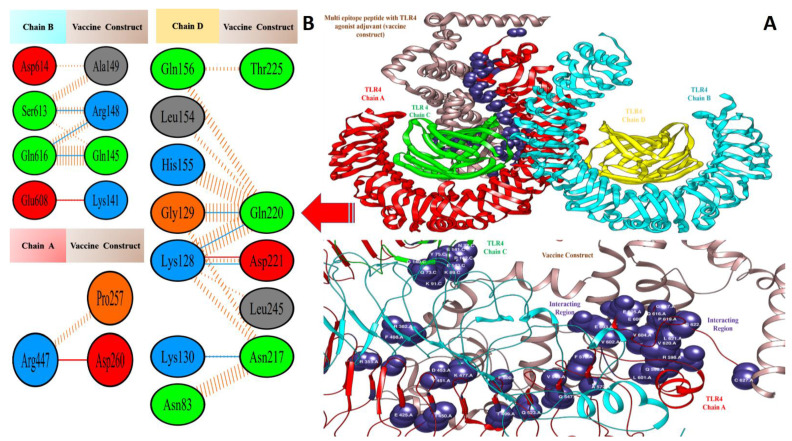
Docked pose of TLR4-agonist vaccine with TLR4: (**A**) every single entity of the complex is self-explanatory. Each chain of the receptor TLR4 is represented in a different colour, i.e., chain A: dark red, chain B: deep sky blue, chain C: green, and chain D: yellow, and the designed vaccine construct TLR4-agonist is represented in dim grey; interacting residues between receptor and vaccine are shown in dark slate blue. (**B**) Residue-residue interaction between vaccine construct and TLR4 chains.

**Figure 8:**
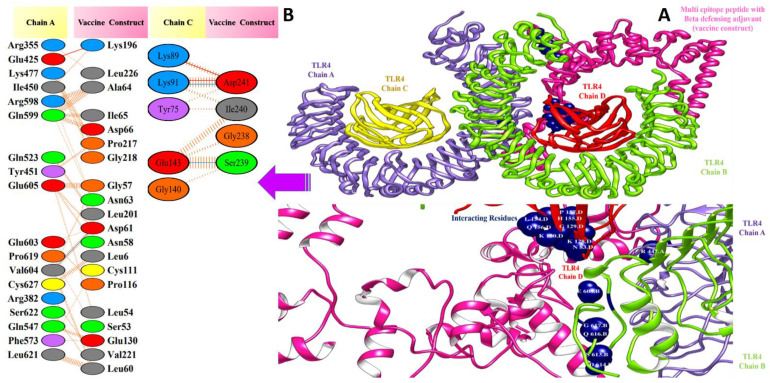
Docked pose of β-defensin vaccine with TLR4: (**A**) every single entity of the complex is self-explanatory. Each chain of the receptor TLR4 is represented in a different colour, i.e., chain A: purple, chain B: green, chain C: yellow, and chain D: dark red, and the designed vaccine construct β-defensin is represented in deep pink; interacting residues between receptor and vaccine are shown in navy blue. (**B**) Residue-residue interaction between vaccine construct and TLR4 chains.

**Figure 9:**
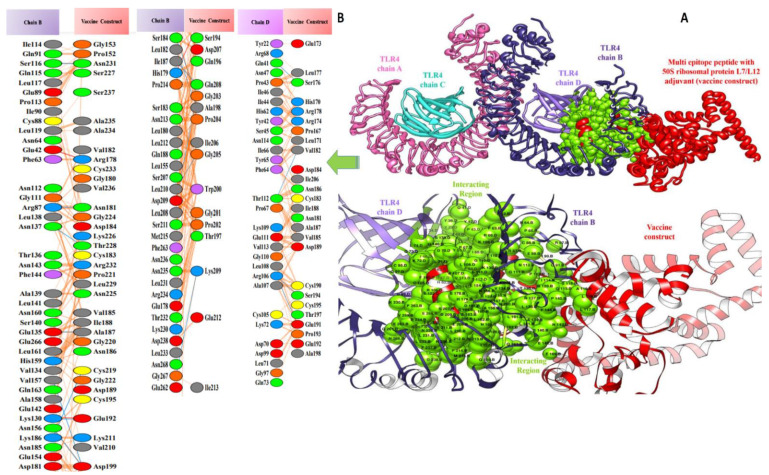
Docked pose of ribosomal protein L7/L12 vaccine with TLR4: (**A**) each entity of the complex is self-explanatory. Each chain of the receptor TLR4 is represented with a different colour, i.e., chain A: magenta, chain B: dark slate blue, chain C: cyan, and chain D: purple, and the designed vaccine construct ribosomal protein L7/L12 vaccine is represented in dark red; interacting residues between receptor and vaccine are shown in green. (**B**) Residue-residue interaction between vaccine construct and TLR4 chains.

In computational vaccine design studies, binding interaction between vaccines and human immune cell receptors is crucial to assure the production of specific cellular and antibody immune responses [72]. Molecular docking is one of the ways to predict the best binding confirmation between vaccine constructs and receptors. To accomplish this, PatchDock [73,74,75], (a blind docking technique) was employed, in which the surface of the TLR4 immune receptor was made accessible for vaccine construct binding. Following that, top solutions were sent to the FireDock web service, which corrects protein flexibility issues that occur during protein–peptide docking and enables high-throughput complex refinement. The refined top ten solutions, ranked by world energy consumption, are shown in Table 3. In the case of the TLR4-agonist vaccine, solution 4 was selected based on the lowest global energy (−29.63 kcal/mol). The major contributions to this global energy were from attractive Van der Waals interactions (−44.67 kcal/mol) and hydrogen bond energy (−5.12 kcal/mol). In the cases of β-defensin and 50S ribosomal protein L7/L12, solution 9 was selected, with global energy of −3.41 kcal/mol (in the case of β-defensin) and −11.03 kcal/mol (in the case of 50S ribosomal protein L7/L12). The three vaccines displayed robust interactions with the receptor molecules and were observed in deep binding. The binding mode and interactions of the vaccines with TLR4 can be seen in Figure 7, Figure 8 and Figure 9.

Valuable insights about designed vaccines binding interactions with TLR4 were obtained via the PDBsum server. It was discovered that hydrogen bonds and hydrophobic interactions are key in the stable binding of the docked complexes. The number of hydrogen bonds between the TLR4 antagonist adjuvant-based vaccine construct and the TLR4 receptor is three. Vaccine residues (ASP61, ASP241, and SER239) were seen in hydrogen bonding with the receptor within a range of 2 Å. The β-defensin adjuvant-based vaccine construct and the TLR4 receptor formed seven hydrogen bonds, and the following residues participated in interaction with the receptor: ARG148, GLN145, ARG148, GLN220, and ASP221. A total of 18 hydrogen bonds were found between the 50S ribosomal protein L7/L12 adjuvant-based vaccine and the TLR4 receptor. Vaccine residues involved in hydrogen bonding are ASN181 ASN231, ALA187, THR228, ASP199, ASP199, ALA198, GLY203, CYS219, GLN208, GLY203, LYS209, SER176, GLU191, SER194, and VAL182. In Figure 7, Figure 8 and Figure 9, interaction maps for bonds are presented alongside each complex to demonstrate intermolecular interactions.

### 3.6. Molecular Dynamics Simulations Analysis

**Figure 10:**
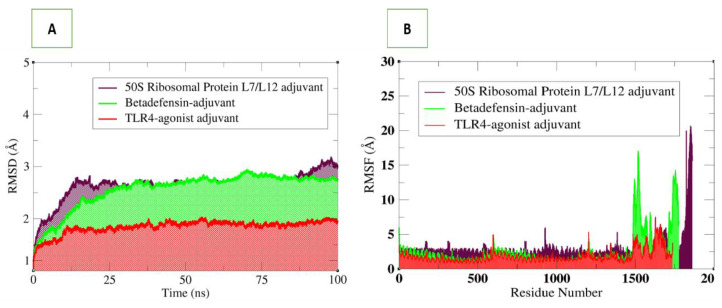
Structural stability analysis of simulated vaccine–receptor complexes: (**A**) RMSD and (**B**) RMSF.

Molecular dynamics simulation is a powerful technique to study the dynamics of docked complexes by allowing the molecules to interact with each other in a fixed time period [43]. The simulation analysis includes root-mean-square deviation (RMSD) which allows superimposition of all simulation snapshots over the initial docked frame. The deviations of the carbon alpha atoms were measured in terms of an angstrom (**Å**) and plotted versus time as shown in Figure 10. The three complexes were found to be highly stable, and the RMSD was found to be within 3 Å. The TLR4 vaccine complex was revealed as the most stable complex, as can be seen in a uniform plot, with very minor fluctuations. The RMSD of the TLR4–vaccine complex was within 2 Å, which is an indication of a highly stable complex. The β-defensin–vaccine complex was be observed in increasing RMSD until 37 ns; then, the system reached a consistent equilibrium till the simulation end. The 50S ribosomal–vaccine complex was detected in continuous dynamics till 25 ns, followed by stable RMSD till 87 ns, and lastly, it experienced a small RMSD surge. Upon visualisation, intermolecular conformation was very stable in all three complexes, and no major deviations were noted, as predicted by RMSD plots. Next, the residue level fluctuations of the complexes were studied via the root-mean-square fluctuations (RMSF) based on carbon alpha atoms. As reflected by RMSD, the fluctuations of the systems were very low. For each system, the vaccine construct residues were noticed in some fluctuations, which may be the reason for vaccine adjustment at the docked site. However, these fluctuations did not affect the overall binding and stability of the systems. The simulation results were further evaluated in terms of stability provided by the disulphide bonds in the vaccine 3D structure. During the dynamics of the complexes, it was observed that disulphide bonds of the vaccines played important roles in giving the vaccine 3D structure more contact and giving more structural strength, allowing the vaccine to be in a stable binding conformation and allowing interactions with the receptors at the docked side.

### 3.7. Salt Bridges between TLR4 and Vaccine Constructs

**Figure 11:**
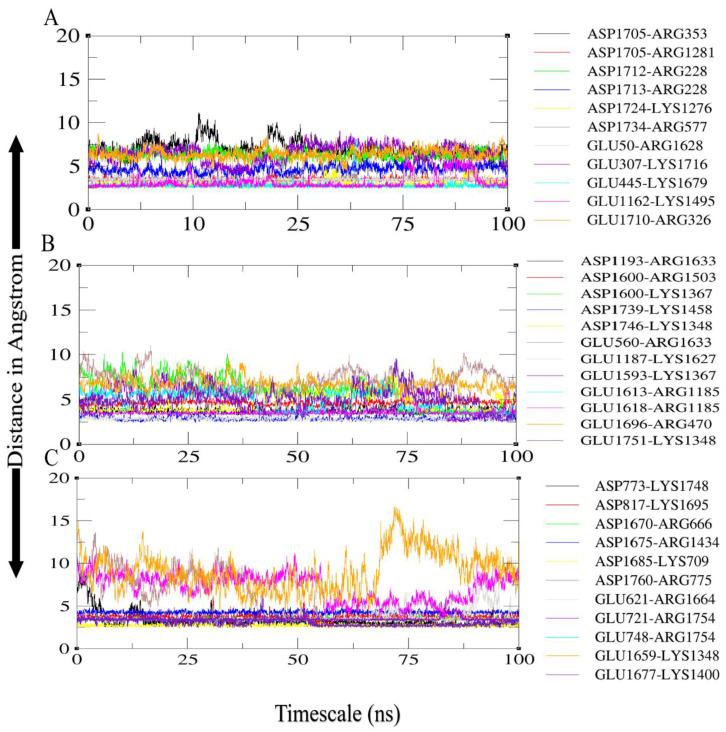
Salt bridges formed between receptor and vaccine: (**A**) TLR4-agonist vaccine with TLR4; (**B**) β-defensin vaccine with TLR4; (**C**) 50S ribosomal protein L7/L12 vaccine with TLR4.

Salt bridges occur between the charged side chains of amino acids in a protein molecule when the pH is neutral. The primary residues involved in these interactions are glutamine and aspartate, which have a negative full electron charge, and arginine and lysine, which have a positive full electron charge. The presence of salt bridges between interacting molecules suggests that the interaction’s stability improved [76]. For the TLR4-agonist vaccine–TLR4 receptor complex, high numbers of salt bridges were calculated within 3.2 Å between TLR4 receptor Arg353, Lys1276, Glu445, Arg577, Glu1162, Glu307, Arg228, Arg228, Arg1281 with the Tlr4-agonist vaccine adjuvant Asp1705, Asp1724, Lys1679, Asp1734, Lys1495, Lys1716, Asp1712, Asp1713, and Asp1705, respectively (Figure 11A). In the case of the β-defensin vaccine–TLR4 complex, receptor residues Arg1185, Lys1348, Lys1367, Arg1185, Lys1367, Glu1187, Asp1193, Glu560, and Lys1458 were involved in salt bridging, with Glu1613, Asp1746, Glu1751, Glu1593, Glu1618, Asp1600, Lys1627, Arg1633, and Asp1739 of the vaccine construct, respectively (Figure 11B). In the case of 50s ribosomal protein L7/L12 vaccine–TLR4 complex, receptor residues Glu748, Arg775, Lys1348, Glu721, Arg1434, Lys709, Glu621, Lys1400, Asp773, Asp817, and Arg666 were involved in salt bridging with Arg1754, Asp1760, Glu1659, Arg1754, Asp1675, Asp1685, Arg1664, Glu1677, Lys1748, Lys1695, and Asp1670 of 50s ribosomal protein L7/L12 vaccine (Figure 11C).

### 3.8. Binding Free Energies Calculation

MM–GBSA and MM–PBSA binding free energies were estimated for the complexes to highlight atomic-level interactions. It was found that Van der Waals energy was the most dominant in the gas phase, with polar solvation energy and overall solvation energy. The electrostatic energy was nonsignificant in its contribution to the net gas phase energy, while nonpolar solvation energy was less favourable.

Overall, the net binding energy of all systems revealed excellent binding which was TLR4-agonist vaccine with TLR4 (MM–GBSA, −1628.47 kcal/mol and MM–PBSA, −37.75 kcal/mol); 50S ribosomal protein L7/L12 vaccine with TLR4 complex (MM–GBSA, −194.62 kcal/mol and MM–PBSA, −150.67 kcal/mol); β-defensin vaccine with TLR4 complex (MM–GBSA, −9.80 kcal/mol and MM–PBSA, −42.34 kcal/mol). The complex binding free energy analysis is given in Table 4.

### 3.9. In Silico Cloning

Sequences of vaccine constructs were reversely translated into sequences of DNA via the JCat server to obtain high expression in *E. coli*. The *E. coli* expression system was selected for the synthesis of recombinant proteins. To ensure that our recombinant vaccine proteins were produced at a high level in the *E. coli* K12 system, we carried out codon optimisation. The GC content value for TLR4-agonist, β-defensin, and 50S ribosomal protein L7/L12 vaccines was 58.36%, 58.30% and 54.35%, respectively. The CAI value for the TLR4-agonist vaccine was 1.0, for β-defensin, it was 0.93, and for 50S ribosomal protein L7/L12 adjuvant vaccine, it was 0.96. All these values were within an acceptable range, indicating greater expression of the vaccines.

### 3.10. Clustering Analysis of Vaccine Constructs

**Figure 12:**
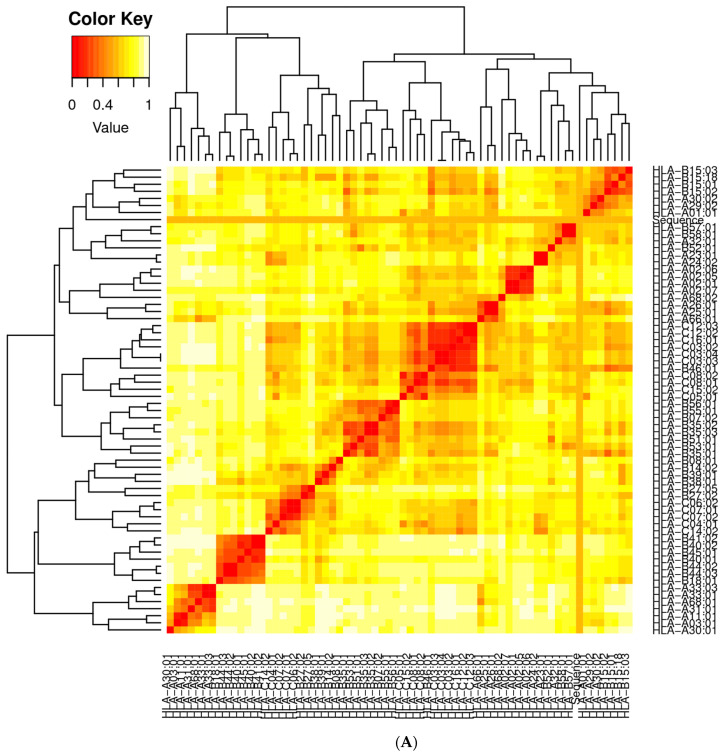

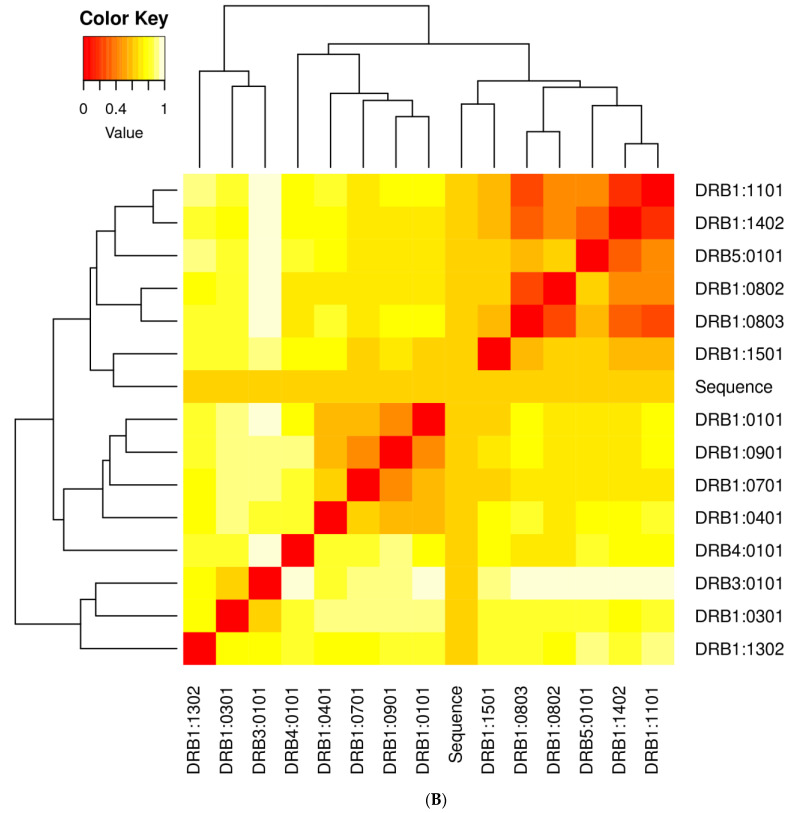
(**A**) Major histocompatibility complex (MHC) I clustering analysis for T cell epitopes; (**B**) major histocompatibility complex (MHC) II clustering analysis for T cell epitopes.

It is essential to find candidates with an optimal affinity for a wide variety of MHC human leukocyte antigen (HLA) alleles while designing vaccines. MHC cluster v2.0 was employed for MHC clustering analysis (http://www.cbs.dtu.dk/services/MHCcluster (accessed on 12 February 2021)) [77]. Figure 12A,B describe the schematic representation of MHC class I and II clustering analysis of vaccine constructs, respectively.

### 3.11. Computational Immune Simulation

**Figure 13:**
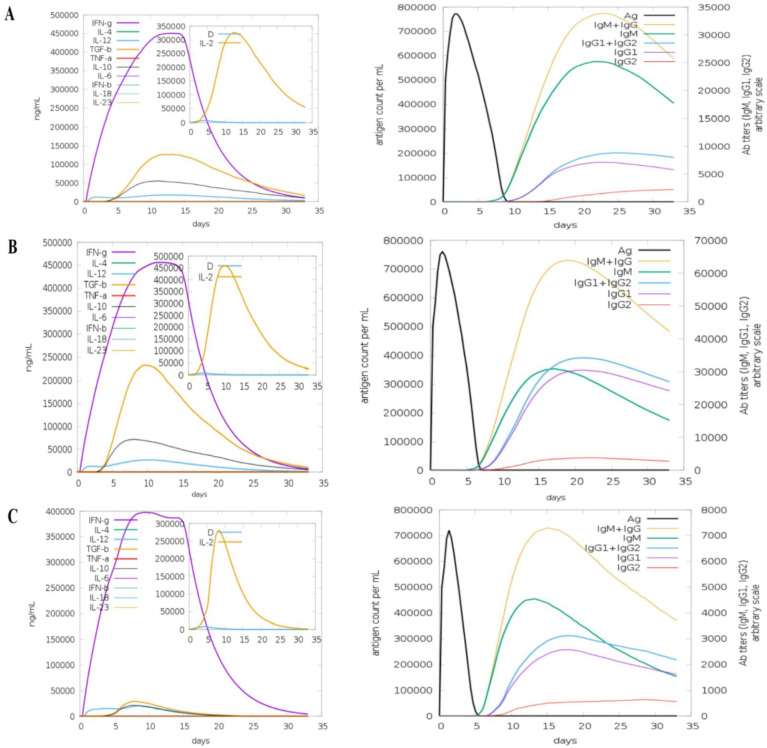
C-Immune Simulation: (**A**) TLR4-agonist, (**B**) β-defensin, and (**C**) 50S ribosomal protein L7/L12 vaccines.

To analyse the immunogenic profiles of the designed vaccines, the C-IMMSIM server was used. All primary, secondary, and tertiary immune responses were quite significant contributors to immunity to vaccine antigens. In addition to this, a combination of IgM + IgG was observed in the higher titres, followed by IgG1 + IgG2 and IgM, as shown in Figure 13. In particular, elevated B cell isotopes were formed in response to the vaccines, which resulted in the formation of memory cells. The strong responses of cytokines and interleukins were also analysed and demonstrated that the vaccine constructs induce high levels of IFN-γ and IL-2.

## 4. Concluding Remarks

No licensed vaccine is currently available against hantavirus infection, though several are under clinical investigation. Inactivated hantavirus vaccines are approved for human use in Korea and China, but no such vaccines are licensed in the US or Europe. The present clinical trials of inactivated hantavirus vaccine and those of DNA vaccines in the US are summarised by Liu et al. [78]. Antigenic, virulent, nontoxigenic, nonallergic, and good DRB*0101 binder epitopes were used to design in silico multi-epitopes based vaccines against hantaviruses in the present study. Docking studies predicted that the designed vaccine constructs would have a high binding affinity with the TLR4 immune cell receptor for stimulating cellular and humoral immune responses. Furthermore, molecular dynamics simulations revealed that the intermolecular interactions are quite stable. The docking results were validated by calculating binding free energy. Despite the fact that these in silico predictions are extremely promising, this study’s primary flaw is the lack of experimental validations, which leaves experimentalists free to assess the immune protection efficiency of the proposed vaccine design.

## Figures and Tables

**Table 1:** Shortlisted epitopes for design of multi-epitopes vaccine constructs.

Epitopes	Host (s)	Allergenicity	Antigenicity	Solubility	IFN	Toxicity	Virulence	
DMRNTIMASKTVGTA	Human	Nonallergen	0.95	Soluble	Positive	Nontoxic	Virulent	1.04
DTKPTDPTGIEPDDHLKERSSLRYGNVLDVNAIDIEEPSGQTADW	Vole	Nonallergen	0.88	Soluble	Positive	Nontoxic	Virulent	0.99
IDQKVKEISNQEPL	Human, rabbit, vole	Nonallergen	0.84	Soluble	Positive	Nontoxic	Virulent	1.02
NKSTLQNRRAAVS	Human Mouse	Nonallergen	0.88	soluble	Positive	Nontoxic	Virulent	1.05
NVLDVNAIDIEEPS	Human, Rabbit, Vole	Nonallergen	0.75	soluble	Positive	Nontoxic	Virulent	1.05
KEKSSLRYGNVLDVN	Mouse,	Nonallergen	1.06	Soluble	Positive	Nontoxic	Virulent	1.02
RNTIMASKTVGTAE	Human, rabbit, vole	Nonallergen	0.73	soluble	Positive	Nontoxic	Virulent	1.03
GKNIGQDRDPTGVEPGDHLKERSALSYGNTLDLNSLDID	Mouse	Nonallergen	0.80	soluble	Positive	Nontoxic	Virulent	0.99
VDPTGLEPDDHLK	Human Mouse	Nonallergen	0.94	soluble	Positive	Nontoxic	Virulent	1.05
SIDLEEPSGQTADWK	Human	Nonallergen	0.70	soluble	Positive	Nontoxic	Virulent	1.05

**Table 2:** Physical parameters for validation of final subunit vaccine constructs.

**TLR4-Agonist**						
**Model**	**GDT-HA**	**RMSD**	**MolProbity**	**Clash Score**	**Poor Rotamers**	**Rama Favoured**
Initial	1	0	3.692	104.6	4.9	83.6
MODEL 1	0.9225	0.499	2.123	15.4	0.5	93.4
MODEL 2	0.9254	0.494	2.184	15.4	0.5	91.8
MODEL 3	0.9254	0.491	2.163	15.7	0.5	92.6
MODEL 4	0.9283	0.471	2.134	14.1	0.5	92.2
MODEL 5	0.9176	0.507	2.061	13.8	0.5	93.8
**β-Defensin**						
**Model**	**GDT-HA**	**RMSD**	**MolProbity**	**Clash Score**	**Poor Rotamers**	**Rama Favoured**
Initial	1	0	3.707	101.6	5.1	82.7
MODEL 1	0.8978	0.55	2.164	17.9	0	93.8
MODEL 2	0.8995	0.54	2.153	16.7	0.9	93.4
MODEL 3	0.8952	0.559	2.169	18.1	0.4	93.8
MODEL 4	0.8918	0.557	2.164	17.9	0.9	93.8
MODEL 5	0.9012	0.545	2.18	17.9	0.4	93.4
**50S Ribosomal Protein L7/L12**						
**Model**	**GDT-HA**	**RMSD**	**MolProbity**	**Clash Score**	**Poor Rotamers**	**Rama Favoured**
Initial	1	0	3.449	98.8	3.7	89.8
MODEL 1	0.9162	0.495	1.99	14.8	0.7	95.5
MODEL 2	0.9182	0.492	2.1	14.8	1.4	95.5
MODEL 3	0.9162	0.493	1.965	13.9	0.7	95.5
MODEL 4	0.9195	0.487	1.96	13.7	0.3	95.5
MODEL 5	0.9182	0.498	1.909	12	0.3	95.5

cla3.5 disulphide engineering of designed vaccine constructs.

**Table 3:** Top 10 docked complexes of designed vaccines with TLR4. The values can be interpreted in kcal/mol.

**TLR4−Agonist**						
**Rank**	**Solution Number**	**Global Energy**	**Attractive VdW**	**Repulsive VdW**	**Atomic Contact Energy**	**Hydrogen Bond Energy**
		↓				
1	4	−29.63	−44.67	41.32	4.84	−5.12
2	7	6.71	−15.14	12.21	−1.23	−1.20
3	3	16.63	−23.10	24.78	8.02	−2.98
4	8	30.40	−17.64	22.04	12.26	−2.67
5	2	79.31	−8.74	81.87	2.58	−0.84
6	10	83.47	−49.20	155.60	15.30	−4.87
7	9	107.83	−42.49	146.03	17.89	−5.37
8	5	121.17	−32.38	193.07	6.66	−2.80
9	1	432.16	−30.72	570.71	4.75	−2.82
10	6	4149.68	−63.85	5310.77	8.60	−4.70
**β−Defensin**						
**Rank**	**Solution Number**	**Global Energy**	**Attractive VdW**	**Repulsive VdW**	**Atomic Contact Energy**	**Hydrogen Bond Energy**
		↓				
1	9	−3.41	−5.43	1.69	2.98	−2.97
2	7	10.42	−3.55	0.40	1.50	0.00
3	2	10.51	−58.47	80.50	20.95	−10.00
4	4	31.11	−3.73	0.00	4.15	0.00
5	6	364.44	−51.20	547.20	5.50	−10.07
6	1	615.86	−53.65	872.84	4.94	−1.23
7	10	918.64	−57.19	1206.60	8.00	−9.37
8	3	1371.21	−72.88	1860.69	4.42	−14.33
9	5	1617.02	−83.97	2179.88	17.45	−18.53
10	8	4445.60	−95.90	5699.42	15.36	−13.35
**50S Ribosomal Protein L7/L12**						
**Rank**	**Solution Number**	**Global Energy**	**Attractive VdW**	**Repulsive VdW**	**Atomic Contact Energy**	**Hydrogen Bond Energy**
		↓				
1	9	−11.03	−9.67	7.15	0.93	−1.31
2	4	−0.43	−3.27	1.17	2.48	−1.33
3	5	2.61	−4.54	6.50	2.57	−0.73
4	7	13.94	−10.63	5.23	2.94	−0.45
5	10	18.08	−1.80	0.00	3.89	−0.95
6	2	54.13	−39.14	116.61	8.41	−2.65
7	8	108.38	−40.95	195.97	11.18	−6.06
8	3	192.03	−16.85	216.91	14.88	−2.43
9	1	3011.31	−37.19	3794.70	6.44	−3.85
10	6	6854.97	−119.49	8726.07	23.31	−21.54

**Table 4:** Binding free energies estimated for complexes. The values are given in kcal/mol.

MMGBSA				MMPBSA			
**TLR4-Agonist Vaccine with TLR4**							
**Energy Component**	**Average**	**Std Dev**	**Err. of Mean**	**Energy Component**	**Average**	**Std Dev**	**Err. of Mean**
Van der Waals Energy	−220.7	35.5	3.5	Van der Waals Energy	−220.7	35.5	3.5
Electrostatic Energy	1798.2	82.3	8.2	Electrostatic Energy	1798.2	82.3	8.2
Gas-Phase Energy	1577.5	55.4	5.5	Gas-Phase Energy	1577.5	55.4	5.5
Solvation Energy	−1628.4	56.6	5.66	Solvation Energy	−1615.3	54.0	5.4
Total	−1628.4	13.3	1.33	Total	−37.7	15.9	1.5
**β-Defensin Vaccine with TLR4**							
Van der Waals Energy	−90.2	17.1	1.7	Van der Waals Energy	−90.2	17.1	1.7
Electrostatic Energy	790.7	74.6	7.4	Electrostatic Energy	790.7	74.6	7.4
Gas-Phase Energy	700.4	59.4	5.9	Gas-Phase Energy	700.4	59.4	5.9
Solvation Energy	−710.29	53.6	5.	Solvation Energy	−742.8	52.5	5.2
Total	−9.8	8.1	0.8	Total	−42.3	10.3	1.0
**50S Ribosomal Protein L7/L12 Vaccine with TLR4**							
Van der Waals Energy	−361.1	35.6	3.5	Van der Waals Energy	−361.1	35.6	3.5
Electrostatic Energy	1723.3	52.5	5.2	Electrostatic Energy	1723.3	52.5	5.2
Gas-Phase Energy	1362.1	45.7	4.5	Gas-Phase Energy	1362.1	45.7	4.5
Solvation Energy	−1556.8	48.6	4.8	Solvation Energy	−1512.8	72.3	7.2
Total	−194.6	40.9	4.0	Total	−150.6	65.6	6.5

## Data Availability

Not applicable.

## Supplementary Materials

The following supporting information can be downloaded at: https://www.mdpi.com/article/10.3390/vaccines10030378/s1, Table S1: Country-wise population coverage of each epitopes; Table S2: Selected epitopes; Table S3: Pair of residues, which were mutated to cysteine amino acid.
