# Supplementary material for: Design of a Multi-Epitopes Vaccine against Hantaviruses: An Immunoinformatics and Molecular Modelling Approach
Source: Vaccines (Basel). 2022 Feb 28;10(3):378. doi: 10.3390/vaccines10030378 (PMC8953224; doi:10.3390/vaccines10030378)
Supplement: Supplementary file 1 [file vaccines-10-00378-s001.zip › vaccines-1595474-supplementary.pdf]

Table S1. Country-wise population coverage of each epitopes.

population/area	Class I	Class II	Class combined
	Coverage		
Algeria	0.00%	77.15%	77.15%
Algeria Arab	0.00%	77.15%	77.15%
American Samoa	98.75%	0.00%	98.75%
American Samoa Polynesian	98.75%	0.00%	98.75%
Argentina	97.50%	62.67%	99.07%
Argentina Amerindian	97.50%	45.78%	98.64%
Argentina Caucasoid	0.00%	80.65%	80.65%
Australia	94.51%	33.15%	96.33%
Australia Australian Aborigines	87.31%	33.15%	91.52%
Australia Caucasoid	99.94%	0.00%	99.94%
Austria	99.91%	93.34%	99.99%
Austria Caucasoid	99.91%	93.34%	99.99%
Belarus	0.00%	43.81%	43.81%
Belarus Caucasoid	0.00%	43.81%	43.81%
Belgium	99.39%	79.39%	99.87%
Belgium Caucasoid	99.39%	79.39%	99.87%
Bolivia	0.00%	77.82%	77.82%
Bolivia Amerindian	0.00%	77.82%	77.82%
Borneo	0.00%	49.02%	49.02%
Borneo Austronesian	0.00%	49.02%	49.02%
Brazil	96.10%	63.80%	98.59%
Brazil Amerindian	93.24%	48.60%	96.52%
Brazil Caucasoid	99.32%	84.39%	99.89%
Brazil Mixed	97.02%	77.50%	99.33%
Brazil Mulatto	0.00%	74.09%	74.09%
Bulgaria	99.42%	57.23%	99.75%
Bulgaria Caucasoid	99.52%	57.23%	99.79%
Bulgaria Other	99.58%	0.00%	99.58%
Burkina Faso	67.18%	0.00%	67.18%
Burkina Faso Black	67.18%	0.00%	67.18%

Cameroon	89.72%	49.87%	94.85%
Cameroon Black	89.72%	49.87%	94.85%
Canada	0.00%	38.41%	38.41%
Canada Amerindian	0.00%	38.41%	38.41%
Cape Verde	99.18%	80.38%	99.84%
Cape Verde Black	99.18%	80.38%	99.84%
Central Africa	86.04%	62.71%	94.79%
Central African Republic	28.02%	82.69%	87.54%
Central African Republic Black	28.02%	82.69%	87.54%
Central America	7.76%	49.91%	53.80%
Chile	95.94%	67.08%	98.66%
Chile Amerindian	99.63%	72.65%	99.90%
Chile Mixed	90.09%	52.65%	95.31%
China	94.57%	59.99%	97.83%
China Oriental	94.57%	59.99%	97.83%
Colombia	8.36%	54.02%	57.86%
Colombia Amerindian	0.00%	47.40%	47.40%
Colombia Black	3.65%	65.25%	66.51%
Colombia Mestizo	14.07%	56.31%	62.45%
Congo	0.00%	68.66%	68.66%
Congo Black	0.00%	68.66%	68.66%
Cook Islands	0.00%	78.59%	78.59%
Cook Islands Polynesian	0.00%	78.59%	78.59%
Costa Rica	0.00%	24.31%	24.31%
Costa Rica Mestizo	0.00%	24.31%	24.31%
Croatia	99.79%	66.71%	99.93%
Croatia Caucasoid	99.79%	66.71%	99.93%
Cuba	98.92%	85.48%	99.84%
Cuba Caucasoid	99.05%	0.00%	99.05%
Cuba Mixed	0.00%	85.48%	85.48%
Cuba Mulatto	98.74%	0.00%	98.74%
Czech Republic	99.08%	86.21%	99.87%
Czech Republic Caucasoid	99.08%	88.76%	99.90%
Czech Republic Other	0.00%	64.14%	64.14%

Denmark	0.00%	88.98%	88.98%
Denmark Caucasoid	0.00%	88.98%	88.98%
East Africa	90.78%	68.30%	97.08%
East Asia	98.18%	81.82%	99.67%
Ecuador	77.35%	52.17%	89.16%
Ecuador Amerindian	77.35%	52.17%	89.16%
England	99.93%	93.48%	100.00%
England Caucasoid	99.93%	93.48%	100.00%
Equatorial Guinea	0.00%	47.58%	47.58%
Equatorial Guinea Black	0.00%	47.58%	47.58%
Ethiopia	0.00%	83.00%	83.00%
Ethiopia Black	0.00%	83.00%	83.00%
Europe	99.68%	85.83%	99.96%
Fiji	0.00%	79.87%	79.87%
Fiji Melanesian	0.00%	79.87%	79.87%
Finland	99.99%	51.14%	100.00%
Finland Caucasoid	99.99%	51.14%	100.00%
France	99.80%	88.54%	99.98%
France Caucasoid	99.80%	88.54%	99.98%
Gabon	0.00%	41.78%	41.78%
Gabon Black	0.00%	41.78%	41.78%
Georgia	98.32%	75.05%	99.58%
Georgia Caucasoid	98.94%	75.05%	99.74%
Georgia Kurd	98.19%	0.00%	98.19%
Germany	99.93%	91.14%	99.99%
Germany Caucasoid	99.93%	91.14%	99.99%
Greece	0.00%	66.92%	66.92%
Greece Caucasoid	0.00%	66.92%	66.92%
Guatemala	7.76%	49.16%	53.11%
Guatemala Amerindian	7.76%	49.16%	53.11%
Guinea-Bissau	96.39%	71.16%	98.96%
Guinea-Bissau Black	96.39%	71.16%	98.96%
Hong Kong	96.05%	0.00%	96.05%
Hong Kong Oriental	96.05%	0.00%	96.05%
India	89.41%	74.99%	97.35%

India Asian	89.41%	74.99%	97.35%
Indonesia	86.79%	47.84%	93.11%
Indonesia Austronesian	86.79%	47.84%	93.11%
Iran	97.50%	64.22%	99.11%
Iran Kurd	0.00%	55.78%	55.78%
Iran Persian	97.50%	65.72%	99.14%
Ireland Northern	99.94%	94.65%	100.00%
Ireland Northern Caucasoid	99.94%	94.65%	100.00%
Ireland South	99.93%	93.15%	100.00%
Ireland South Caucasoid	99.93%	93.15%	100.00%
Israel	89.39%	68.79%	96.69%
Israel Arab	94.42%	67.51%	98.19%
Israel Jew	93.75%	69.65%	98.10%
Italy	99.03%	85.90%	99.86%
Italy Caucasoid	99.03%	85.90%	99.86%
Ivory Coast	67.75%	0.00%	67.75%
Ivory Coast Black	67.75%	0.00%	67.75%
Jamaica	0.00%	27.41%	27.41%
Jamaica Black	0.00%	27.41%	27.41%
Japan	98.63%	74.83%	99.66%
Japan Oriental	98.63%	74.83%	99.66%
Jordan	90.83%	52.88%	95.68%
Jordan Arab	90.83%	52.88%	95.68%
Kenya	89.56%	0.00%	89.56%
Kenya Black	89.56%	0.00%	89.56%
Kiribati	0.00%	10.89%	10.89%
Kiribati Micronesian	0.00%	10.89%	10.89%
Korea; South	98.21%	85.32%	99.74%
Korea; South Oriental	98.21%	85.32%	99.74%
Lebanon	0.00%	70.46%	70.46%
Lebanon Arab	0.00%	70.46%	70.46%
Macedonia	26.72%	66.53%	75.47%
Macedonia Caucasoid	26.72%	66.53%	75.47%
Malaysia	81.38%	57.99%	92.18%
Malaysia Austronesian	63.18%	55.38%	83.57%

Malaysia Oriental	87.82%	70.35%	96.39%
Mali	96.02%	0.00%	96.02%
Mali Black	96.02%	0.00%	96.02%
Martinique	22.56%	74.51%	80.26%
Martinique Black	22.56%	74.51%	80.26%
Mexico	97.97%	55.04%	99.09%
Mexico Amerindian	99.87%	42.59%	99.93%
Mexico Mestizo	98.13%	68.51%	99.41%
Mongolia	95.31%	81.85%	99.15%
Mongolia Oriental	95.31%	81.85%	99.15%
Morocco	98.63%	83.44%	99.77%
Morocco Arab	99.32%	85.07%	99.90%
Morocco Caucasoid	97.94%	79.75%	99.58%
Nauru	0.00%	38.66%	38.66%
Nauru Micronesian	0.00%	38.66%	38.66%
Netherlands	0.00%	83.44%	83.44%
Netherlands Caucasoid	0.00%	83.44%	83.44%
New Caledonia	98.18%	81.41%	99.66%
New Caledonia Melanesian	98.18%	81.41%	99.66%
New Zealand	0.00%	84.46%	84.46%
New Zealand Polynesian	0.00%	84.46%	84.46%
Niue	0.00%	77.82%	77.82%
Niue Polynesian	0.00%	77.82%	77.82%
North Africa	96.03%	75.06%	99.01%
North America	99.06%	87.89%	99.89%
Northeast Asia	94.70%	59.99%	97.88%
Norway	0.00%	94.71%	94.71%
Norway Caucasoid	0.00%	94.71%	94.71%
Oceania	94.71%	59.87%	97.88%
Oman	99.69%	0.00%	99.69%
Oman Arab	99.69%	0.00%	99.69%
Pakistan	97.09%	1.18%	97.13%
Pakistan Asian	96.75%	1.45%	96.79%
Pakistan Mixed	97.73%	0.00%	97.73%
Papua New Guinea	97.92%	69.15%	99.36%

Papua New Guinea Melanesian	97.92%	69.15%	99.36%
Paraguay	0.00%	4.90%	4.90%
Paraguay Amerindian	0.00%	4.90%	4.90%
Peru	99.99%	49.87%	100.00%
Peru Amerindian	99.99%	49.87%	100.00%
Philippines	94.98%	28.56%	96.41%
Philippines Austronesian	94.98%	28.56%	96.41%
Poland	99.77%	84.46%	99.96%
Poland Caucasoid	99.77%	84.46%	99.96%
Portugal	98.72%	78.00%	99.72%
Portugal Caucasoid	98.72%	78.00%	99.72%
Romania	99.67%	0.00%	99.67%
Romania Caucasoid	99.67%	0.00%	99.67%
Russia	99.27%	77.62%	99.84%
Russia Caucasoid	3.96%	88.52%	88.97%
Russia Mixed	5.05%	0.00%	5.05%
Russia Other	99.98%	85.01%	100.00%
Russia Siberian	99.43%	78.83%	99.88%
Rwanda	24.87%	62.79%	72.05%
Rwanda Black	24.87%	62.79%	72.05%
Samoa	0.00%	80.86%	80.86%
Samoa Polynesian	0.00%	80.86%	80.86%
Sao Tome and Principe	97.02%	66.50%	99.00%
Sao Tome and Principe Black	97.02%	66.50%	99.00%
Saudi Arabia	98.26%	80.14%	99.65%
Saudi Arabia Arab	98.26%	80.14%	99.65%
Scotland	65.34%	90.82%	96.82%
Scotland Caucasoid	65.34%	90.82%	96.82%
Senegal	95.58%	30.28%	96.92%
Senegal Black	95.58%	30.28%	96.92%
Serbia	73.37%	0.00%	73.37%
Serbia Caucasoid	73.37%	0.00%	73.37%
Singapore	92.66%	65.78%	97.49%

Singapore Austronesian	90.55%	65.78%	96.77%
Singapore Oriental	94.81%	0.00%	94.81%
Slovakia	0.00%	18.28%	18.28%
Slovakia Caucasoid	0.00%	18.28%	18.28%
Slovenia	0.00%	84.85%	84.85%
Slovenia Caucasoid	0.00%	84.85%	84.85%
South Africa	93.03%	32.10%	95.27%
South Africa Black	91.96%	32.10%	94.54%
South Africa Other	97.61%	0.00%	97.61%
South America	88.30%	58.59%	95.15%
South Asia	94.73%	75.38%	98.70%
Southeast Asia	94.56%	56.98%	97.66%
Southwest Asia	92.50%	43.93%	95.79%
Spain	87.52%	80.51%	97.57%
Spain Caucasoid	87.52%	80.84%	97.61%
Spain Other	0.00%	6.30%	6.30%
Sri Lanka	52.39%	0.00%	52.39%
Sri Lanka Asian	52.39%	0.00%	52.39%
Sudan	93.78%	60.56%	97.55%
Sudan Arab	70.21%	0.00%	70.21%
Sudan Black	2.19%	0.00%	2.19%
Sudan Mixed	94.39%	60.56%	97.79%
Sweden	99.99%	88.07%	100.00%
Sweden Caucasoid	99.99%	88.07%	100.00%
Taiwan	97.77%	67.88%	99.29%
Taiwan Oriental	97.77%	67.88%	99.29%
Thailand	91.21%	63.90%	96.83%
Thailand Oriental	91.21%	63.90%	96.83%
Tokelau	0.00%	55.11%	55.11%
Tokelau Polynesian	0.00%	55.11%	55.11%
Tonga	0.00%	71.91%	71.91%
Tonga Polynesian	0.00%	71.91%	71.91%
Tunisia	97.52%	74.26%	99.36%
Tunisia Arab	97.52%	74.97%	99.38%
Tunisia Berber	0.00%	74.47%	74.47%

Turkey	44.80%	76.19%	86.85%
Turkey Caucasoid	44.80%	76.19%	86.85%
Uganda	94.87%	0.00%	94.87%
Uganda Black	94.87%	0.00%	94.87%
Ukraine	0.00%	50.64%	50.64%
Ukraine Caucasoid	0.00%	50.64%	50.64%
United Arab Emirates	3.37%	32.92%	35.19%
United Arab Emirates Arab	3.37%	32.92%	35.19%
United States	99.10%	88.10%	99.89%
United States Amerindian	99.67%	42.79%	99.81%
United States Asian	97.46%	78.84%	99.46%
United States Austronesian	0.00%	58.09%	58.09%
United States Black	97.11%	71.50%	99.18%
United States Caucasoid	99.83%	90.15%	99.98%
United States Hispanic	98.92%	72.95%	99.71%
United States Mestizo	99.23%	72.23%	99.79%
United States Polynesian	99.57%	73.18%	99.88%
Venezuela	90.04%	3.01%	90.34%
Venezuela Amerindian	90.07%	0.00%	90.07%
Venezuela Caucasoid	11.45%	0.00%	11.45%
Venezuela Mestizo	9.75%	0.00%	9.75%
Venezuela Mixed	0.00%	3.17%	3.17%
Vietnam	91.82%	54.44%	96.27%
Vietnam Oriental	91.82%	54.44%	96.27%
Wales	1.00%	0.00%	1.00%
Wales Caucasoid	1.00%	0.00%	1.00%
West Africa	95.49%	65.23%	98.43%
West Indies	98.98%	69.22%	99.69%
Zambia	98.10%	0.00%	98.10%
Zambia Black	98.10%	0.00%	98.10%
Zimbabwe	93.79%	68.30%	98.03%
Zimbabwe Black	93.79%	68.30%	98.03%
Average	62.93	55.21	82.86
Standard deviation	43.71	30.27	25.28

Table S2. Selected epitopes.

	IEDB ID		Protein Names	Assay Type Category
1	101	AAECPFLPKPKVA	nucleoprotein,	B Cell
2	432	AASGDPTSPDNIDS	nucleocapsid protein,	B Cell
3	638	ADAVSRKKMD	N protein,nucleocapsid protein,	B Cell
4	637	ADAVSRKKMD	nucleocapsid protein,	B Cell
5	677	ADIDKLIDYAASGD	nucleocapsid protein,	B Cell
6	723	ADLVAAQKLATKP	nucleoprotein,	B Cell
7	881	AEEKLKKKSSFYQS	nucleocapsid protein,	B Cell
8	883	AEELTPGRFRTIVC	nucleocapsid protein,	B Cell
9	1146	AERAVEVDPDDVNK	nucleocapsid protein,	B Cell
10	1299	AFFSILQDMRNTIM	nucleocapsid protein,	B Cell
11	1872	AIDIEEPSGQ	N protein,nucleocapsid protein,	B Cell
12	2621	ALIDQKVKEI	nucleocapsid protein,	B Cell
13	4074	AQSTMKAEELTPGR	nucleocapsid protein,	B Cell
14	4220	ARQKLKDAER	N protein,nucleocapsid protein,	B Cell
15	4219	ARQKLKDAER	nucleocapsid protein,	B Cell
16	4227	ARQQTVSALE	N protein,nucleocapsid protein,	B Cell
17	4226	ARQQTVSALE	nucleocapsid protein,	B Cell
18	4228	ARQQTVSALEDKLA	nucleocapsid protein,	B Cell
19	5311	AVEVDPDDVN	N protein,nucleocapsid protein,	B Cell
20	5310	AVEVDPDDVN	nucleocapsid protein,	B Cell
21	5312	AVEVDPDDVNKNTL	nucleocapsid protein,	B Cell
22	6762	CPFIKPEVKPGTPA	nucleocapsid protein,	B Cell
23	6764	CPFLPKPKVASEAFMSTNKM	nucleoprotein,	B Cell
24	7496	DAEKAVEVDPDDV	nucleoprotein,	B Cell
25	7671	DAVSRKKMDTKPTD	nucleocapsid protein,	B Cell
26	7885	DDVNKNTLQARQQT	nucleocapsid protein,	B Cell
27	8760	DIQEDITRHE	nucleocapsid protein,	B Cell
28	8761	DIQEEITRHE	N protein,nucleocapsid protein,	B Cell
29	8804	DITRHEQQLIVARQ	nucleocapsid protein,	B Cell
30	8918	DKLADYKRRM	N protein,nucleocapsid protein,	B Cell
31	8917	DKLADYKRRM	nucleocapsid protein,	B Cell
32	8927	DKLIDYAASGDPTS	nucleocapsid protein,	B Cell
33	9399	DMRNTIMASK	nucleocapsid protein,	B Cell
34	9401	DMRNTIMASKTVGTA	nucleocapsid protein,	B Cell
35	9472	DNIDSPNAPWVFAC	nucleocapsid protein,	B Cell
36	9789	DPTSPDDIES	N protein,	B Cell
37	10387	DTKPTDPTGIEPDDHLKERSSLRYGNVLDVNAIDIEEPSGQTADW	nucleocapsid protein,	B Cell
38	10677	DVNAIDIEEPSGQT	nucleocapsid protein,	B Cell
39	10685	DVNKSTLQARQQTVSALEDKLADYKRRMADAVSRKKMDTKPTDPT	nucleocapsid protein,	B Cell
40	10821	DWPERIRDFM	N protein,	B Cell
41	10824	DWSERIREFM	nucleocapsid protein,	B Cell
42	10843	DWYTIGVYVIGFTL	nucleocapsid protein,	B Cell
43	11353	EDINGIRRPK	N protein,nucleocapsid protein,	B Cell
44	11354	EDINGIRRPKHLYV	nucleocapsid protein,	B Cell
45	11370	EDKLADYKRRMADA	nucleocapsid protein,	B Cell
46	11724	EEKLKKKSSF	nucleocapsid protein,	B Cell
47	11772	EELTPGRFRT	N protein,nucleocapsid protein,	B Cell
48	12050	EFMEKECPFIKPEV	nucleocapsid protein,	B Cell
49	12440	EIEMLKRNKIYFMQ	nucleocapsid protein,	B Cell
50	12659	EKECPFIKPEVKPG	nucleocapsid protein,	B Cell
51	12660	EKECPFIKPEVKPGTPAQEIEMLKRNKIYF	nucleocapsid protein,	B Cell
52	13063	ELGAFFSILQ	N protein,nucleocapsid protein,	B Cell
53	13064	ELGAFFSILQDMR	nucleoprotein,	B Cell

54	*13065*	ELGAFFSILQDMRN	nucleocapsid protein,	B Cell
55	*987*	AEIESATLF	nucleocapsid protein,	MHC Binding
56	*1044*	AELGAFFSI	nucleocapsid protein,	MHC Binding
57	*1357*	AFMATNKAY	nucleocapsid protein,	MHC Binding
58	*3989*	AQKLATKPV	nucleocapsid protein,	MHC Binding
59	*5080*	ATPHSVWVF	nucleocapsid protein,	MHC Binding
60	*13137*	ELKRQLADL	nucleocapsid protein,	MHC Binding
61	*13230*	ELQENITAH	nucleocapsid,nucleocapsid protein,	MHC Binding
62	*639*	ADAVSRKKMDTKPTDPT	N protein,	T Cell
63	*2000*	AILQDMRNTI	nucleocapsid protein,	T Cell
64	*5767*	AYFITRQL	nucleocapsid protein,	T Cell
65	*8762*	DIQEEITRHEQQLVVARQKLKD	N protein,	T Cell
66	*10650*	DVKVKEISNQEPLKL	Nucleoprotein,hypothetical protein,nucleocapsid protein,	T Cell
67	*11258*	ECPFIKPEV	nucleocapsid protein,	T Cell
68	*13061*	ELGAFFAI	nucleocapsid protein,	T Cell
69	*13247*	ELRGLAQALIDQKV	nucleocapsid protein,	B Cell
70	*13248*	ELRGLAQALIDQKVK	nucleocapsid protein,	B Cell
71	*13253*	ELRQLAQSLIDTKVKEISNQEPLKL	nucleoprotein,	B Cell
72	*13396*	EMLKRNKIYF	nucleocapsid protein,	B Cell
73	*13501*	ENKGTRIRFK	nucleocapsid protein,	B Cell
74	*13503*	ENKGTRIRFKDDTS	nucleocapsid protein,	B Cell
75	*13763*	EPSGQTADWY	N protein,nucleocapsid protein,	B Cell
76	*14191*	ESKVQDIIDLIDH	nucleoprotein,	B Cell
77	*14751*	EVKPGTPAQEIEML	nucleocapsid protein,	B Cell

78	*15511*	FEDVNGIRRP	N protein,	B Cell
79	*15517*	FEEINGIRKP	nucleocapsid protein,	B Cell
80	*15518*	FEEINGIRRP	nucleocapsid protein,	B Cell
81	*15523*	FEEVNGIRKP	nucleoprotein,	B Cell
82	*15524*	FEEVNGIRRP	nucleocapsid protein,	B Cell
83	*16120*	FHLGDDMDPELRGL	nucleocapsid protein,	B Cell
84	*16229*	FIKPEVKPGT	nucleocapsid protein,	B Cell
85	*17052*	FMLEWGKEMVDHFH	nucleocapsid protein,	B Cell
86	*18951*	GDDMDPELRGLAQA	nucleocapsid protein,	B Cell
87	*19081*	GDPTSPDNIDSPNA	nucleocapsid protein,	B Cell
88	*20769*	GLAQALIDQKVKEI	nucleocapsid protein,	B Cell
89	*21247*	GMAELGAFFSILQD	nucleocapsid protein,	B Cell
90	*22037*	GRFRTIVCGLFPTQ	nucleocapsid protein,	B Cell
91	*22178*	GRQTVKENKGTRIR	nucleocapsid protein,	B Cell
92	*22799*	GTRIRFKDDTSFED	nucleocapsid protein,	B Cell
93	*22999*	GVIGFSFFVK	N protein,nucleocapsid protein,	B Cell
94	*23727*	HEQQLVTARQKLK	nucleoprotein,	B Cell
95	*23728*	HEQQLVVARQKLKDAERAVEVDPDDV	nucleocapsid protein,	B Cell
96	*24209*	HLGDDMDPEL	N protein,nucleocapsid protein,	B Cell
97	*24330*	HLYVSMPTAQ	nucleocapsid protein,	B Cell
98	*25013*	HVADIDKLID	N protein,nucleocapsid protein,	B Cell
99	*25653*	IDQKVKEISNQEPL	nucleocapsid protein,	B Cell
100	*25786*	IEEPSGQTADWYTI	nucleocapsid protein,	B Cell
101	*26225*	IGFSFFVKDWSERI	nucleocapsid protein,	B Cell
102	*26227*	IGFTLPIILKALYM	nucleocapsid protein,	B Cell
103	*26575*	IILKALYMLSTRGR	nucleocapsid protein,	B Cell
104	*27449*	IMASKTVGTA	nucleocapsid protein,	B Cell
105	*27451*	IMASKTVGTAEEKL	nucleocapsid protein,	B Cell
106	*28144*	IQEDITRHEQQLIV	nucleocapsid protein,	B Cell

107	*28268*	IRDFMEKESP	N protein,	B Cell
108	*28291*	IRFKDDTSFEDING	nucleocapsid protein,	B Cell
109	*28389*	IRRPKHLYVS	N protein,nucleocapsid protein,	B Cell
110	*29067*	ITRHEQQLIV	nucleocapsid protein,	B Cell
111	*29068*	ITRHEQQLIV	nucleocapsid protein,	B Cell
112	*29205*	IVARQKLKDAERAV	nucleocapsid protein,	B Cell
113	*29578*	IYFMQRQDVLDKNH	nucleocapsid protein,	B Cell
114	*29883*	KALYMLSTRG	nucleocapsid protein,	B Cell
115	*30105*	KDAERAVEVD	N protein,nucleocapsid protein,	B Cell
116	*30104*	KDAERAVEVD	nucleocapsid protein,	B Cell
117	*30130*	KDDTSFEDINGIRR	nucleocapsid protein,	B Cell
118	*14363*	ETESATLFT	nucleocapsid,nucleocapsid protein,	MHC Binding
119	*16490*	FLAAECPFL	nucleocapsid protein,	MHC Binding
120	*17108*	FMVAWGKEA	Nucleoprotein,hypothetical protein,	MHC Binding
121	*18201*	FVKDWMDRI	nucleocapsid protein,	MHC Binding
122	*18203*	FVKDWMERI	nucleocapsid protein,	MHC Binding
123	*21234*	GLYPAQIKA	Nucleoprotein,hypothetical protein,nucleocapsid protein,	MHC Binding
124	*23092*	GVPELGAFF	nucleocapsid protein,	MHC Binding
125	*23523*	HAEIESATL	nucleocapsid protein,	MHC Binding
126	*23529*	HAETESATL	nucleocapsid,nucleocapsid protein,	MHC Binding
127	*26574*	IILKALYML	nucleocapsid protein,	MHC Binding
128	*27259*	ILQDMRNTI	Nucleoprotein,hypothetical protein,nucleocapsid protein,	MHC Binding, ,T Cell.
129	*28289*	IRFKDDSSF	nucleocapsid protein,	MHC Binding
130	*14852*	EVQDNITL	nucleocapsid protein,	T Cell
131	*15194*	FAILQDMRNT	nucleocapsid protein,	T Cell
132	*15195*	FAILQDMRNTIMASK	nucleocapsid protein,	T Cell
133	*17746*	FSILQDMRNTIMASK	Nucleoprotein,hypothetical protein,nucleocapsid protein,	T Cell
134	*20865*	GLFPTQIQV	nucleocapsid protein,	T Cell
135	*28282*	IREFMEKECPFIKPE	nucleocapsid protein,	T Cell
136	*28390*	IRRPKHLYVSMPTAQST	N protein,	T Cell
137	*30596*	KERSSLRYGN	N protein,nucleocapsid protein,	B Cell
138	*30597*	KERSSLRYGNVLDV	nucleocapsid protein,	B Cell
139	*31133*	KHLYVSMPTAQSTM	nucleocapsid protein,	B Cell
140	*31701*	KKSSFYQSYL	N protein,nucleocapsid protein,	B Cell
141	*32296*	KMDTKPTDPTGIEP	nucleocapsid protein,	B Cell
142	*32617*	KNTLQARQQT	N protein,nucleocapsid protein,	B Cell
143	*32616*	KNTLQARQQT	nucleocapsid protein,	B Cell
144	*34065*	KVKEISNQEPLKI	nucleocapsid protein,	B Cell
145	*34727*	LADYKRRMADAVSR	nucleocapsid protein,	B Cell
146	*35199*	LDKNHVADIDKLID	nucleocapsid protein,	B Cell
147	*35947*	LFPTQIQVRNIMSP	nucleocapsid protein,	B Cell
148	*36816*	LKDAERAVEVDPDD	nucleocapsid protein,	B Cell
149	*39198*	LRRTQSMGIQLDQ	nucleoprotein,	B Cell
150	*39199*	LRRTQSMGIQLDQR	nucleocapsid protein,	B Cell
151	*39251*	LRYGNVLDVN	N protein,nucleocapsid protein,	B Cell

152	*39714*	LSTRGRQTVK	nucleocapsid protein,	B Cell
153	*39849*	LTDIQEDITRHEQQ	nucleocapsid protein,	B Cell
154	*40099*	LTPGRFRTIVCGLF	nucleocapsid protein,	B Cell
155	*41258*	MDPELRGLAQ	N protein,nucleocapsid protein,	B Cell
156	*41259*	MDPELRGLAQALID	nucleocapsid protein,	B Cell
157	*41592*	MGIQLDQRIILLFM	nucleocapsid protein,	B Cell
158	*41644*	MGVIGFSFFVKDWS	nucleocapsid protein,	B Cell
159	*41979*	MLKRNKIYFMQRQD	nucleocapsid protein,	B Cell
160	*42327*	MPTAQSTMKA	nucleocapsid protein,	B Cell
161	*42329*	MPTAQSTMKAEELT	nucleocapsid protein,	B Cell
162	*42408*	MQRQDVLDKNHVAD	nucleocapsid protein,	B Cell
163	*42531*	MSDLTDIQED	nucleocapsid protein,	B Cell
164	*42532*	MSDLTDIQEDITRH	nucleocapsid protein,	B Cell
165	*42533*	MSDLTDIQEDITRHEQQLVVARQKLKDAEKAVEMYPDDVNKNTLQ	nucleocapsid protein,	B Cell,T Cell
166	*42534*	MSDLTDIQEE	N protein,nucleocapsid protein,	B Cell,
167	*42536*	MSDLTDIQEEITRHEQQLVVARQKLKDAERAVEVDPDDVNKSTLQ	nucleocapsid protein,	B Cell
168	*42678*	MSTLQELQENITA	nucleoprotein,	B Cell
169	*42923*	MVDHFHLGDD	N protein,nucleocapsid protein,	B Cell
170	*44002*	NGIRRPKHLYVSMP	nucleocapsid protein,	B Cell
171	*44172*	NHVADIDKLIDYAA	nucleocapsid protein,	B Cell
172	*44293*	NIMSPVMGVIGFSF	nucleocapsid protein,	B Cell
173	*44563*	NKNTLQARQQTVSA	nucleocapsid protein,	B Cell
174	*44602*	NKSTLQNRRAAVS	nucleoprotein,	B Cell
175	*46127*	NTIMASKSVGTAE	nucleoprotein,	B Cell
176	*46417*	NVLDVNAIDIEEPS	nucleocapsid protein,	B Cell
177	*46912*	PAQEIEMLKR	nucleocapsid protein,	B Cell
178	*47072*	PDDHLKERSS	N protein,nucleocapsid protein,	B Cell
179	*47078*	PDDVNKNTLQ	N protein,nucleocapsid protein,	B Cell
180	*47077*	PDDVNKNTLQ	nucleocapsid protein,	B Cell
181	*47765*	PGTPAQEIEMLKRN	nucleocapsid protein,	B Cell
182	*48115*	PKPKVASEAFMST	nucleoprotein,	B Cell
183	*48603*	PNAPWVFACA	N protein,nucleocapsid protein,	B Cell
184	*49388*	PSGQTADWYTIGVY	nucleocapsid protein,	B Cell
185	*49631*	PTGIEPDDHL	N protein,nucleocapsid protein,	B Cell
186	*50307*	QALIDQKVKEISNQ	nucleocapsid protein,	B Cell
187	*50494*	QDMRNTIMASKTVG	nucleocapsid protein,	B Cell
188	*50528*	QDVLDKNHVADIDK	nucleocapsid protein,	B Cell
189	*51313*	QLDQRIILLFMLEW	nucleocapsid protein,	B Cell
190	*51576*	QLVTARQKLKDAEKAVEVDPDDVNKSTLQNRRAAVSTLETKLG	nucleoprotein,	B Cell
191	*30311*	KEAVNHFHL	nucleocapsid protein,	MHC Binding
192	*32130*	KLRKKSSFY	Nucleoprotein,hypothetical protein,nucleocapsid protein,	MHC Binding
193	*32739*	KPKALSEAF	nucleocapsid,nucleocapsid protein,	MHC Binding
194	*32772*	KPKHLYVSM	nucleocapsid protein,	MHC Binding
195	*32777*	KPKVASEAF	nucleocapsid protein,	MHC Binding
196	*39249*	LRYGNVLDV	nucleocapsid,nucleocapsid protein,	MHC Binding
197	*47346*	PELGAFFAI	nucleocapsid protein,	MHC Binding
198	*30485*	KEKSSLRYGNVLDVN	nucleocapsid protein,	T Cell
199	*35267*	LDQKIIIL	nucleocapsid protein,	T Cell
200	*36052*	LGAFFAIL	nucleocapsid protein,	T Cell
201	*39252*	LRYGNVLDVNAIDIEEPSGQTA	N protein,	T Cell

202	*41256*	MDPELREL	nucleocapsid protein,	T Cell
203	*46910*	PAQEIEFLKRNRVYFMTRQDVL	N protein,	T Cell
204	*49914*	PVMGVIGFS	nucleocapsid protein,	T Cell
205	*52058*	QQLIVARQKL	nucleocapsid protein,	B Cell
206	*52059*	QQLIVARQKLKDAE	nucleocapsid protein,	B Cell
207	*52060*	QQLIVARQKLKDAER	nucleocapsid protein,	B Cell
208	*52456*	QSYLRRTQSMGIQL	nucleocapsid protein,	B Cell
209	*52466*	QTADWYTIGVYVIG	nucleocapsid protein,	B Cell
210	*52660*	QTVSALEDKLADYK	nucleocapsid protein,	B Cell
211	*52720*	QVKARNIISPVMG	nucleoprotein,	B Cell
212	*52777*	QVRNIMSPVM	N protein,nucleocapsid protein,	B Cell
213	*52778*	QVRNIMSPVMGVIG	nucleocapsid protein,	B Cell
214	*53905*	RGLAQALIDQ	nucleocapsid protein,	B Cell
215	*53906*	RGLAQSLIDQ	N protein,	B Cell
216	*53967*	RGRQAVKDNKGTRIRFKDDSSFEEVN	nucleoprotein,	B Cell
217	*54047*	RHEQQLIVARQKLK	nucleocapsid protein,	B Cell
218	*54233*	RIREFMEKECPFIK	nucleocapsid protein,	B Cell
219	*54238*	RIRFKDDTSF	N protein,nucleocapsid protein,	B Cell
220	*54237*	RIRFKDDTSF	nucleocapsid protein,	B Cell
221	*54375*	RKKMDTKPTD	nucleocapsid protein,	B Cell
222	*54848*	RMADAVSRKKMDTK	nucleocapsid protein,	B Cell
223	*55007*	RNKIYFMQRQDVLD	nucleocapsid protein,	B Cell
224	*55066*	RNTIMASKTVGTAE	nucleocapsid protein,	B Cell
225	*55436*	RQKLKDAERAVEVD	nucleocapsid protein,	B Cell
226	*55494*	RQTVKENKGT	nucleocapsid protein,	B Cell
227	*55675*	RRPKHLYVSMPTAQ	nucleocapsid protein,	B Cell
228	*55751*	RRTQSMGIQL	N protein,nucleocapsid protein,	B Cell
229	*56089*	RTIVCGLFPTQIQV	nucleocapsid protein,	B Cell
230	*56537*	RYGNVLDVNAIDIE	nucleocapsid protein,	B Cell
231	*56852*	SALEDKLADYKRRM	nucleocapsid protein,	B Cell
232	*57351*	SEAFMSTNKMYFL	nucleoprotein,	B Cell
233	*57767*	SFFVKDWPER	N protein,	B Cell
234	*57770*	SFFVKDWSER	nucleocapsid protein,	B Cell
235	*57771*	SFFVKDWSERIREF	nucleocapsid protein,	B Cell
236	*57957*	SFYQSYLRRTQSMG	nucleocapsid protein,	B Cell
237	*58614*	SILQDMRNTIMASK	nucleocapsid protein,	B Cell
238	*58954*	SKTVGTAEEKLKKK	nucleocapsid protein,	B Cell
239	*60319*	SPVMGVIGFSFFVK	nucleocapsid protein,	B Cell
240	*60714*	SRKKMDTKPTDPTG	nucleocapsid protein,	B Cell
241	*61192*	SSLRYGNVLDVNAI	nucleocapsid protein,	B Cell
242	*61862*	STRGRQTVKENKGT	nucleocapsid protein,	B Cell
243	*62824*	TADWYTIGVY	N protein,nucleocapsid protein,	B Cell
244	*62975*	TAQSTMKAEEITP	nucleoprotein,	B Cell
245	*63215*	TDPTGIEPDDHLKE	nucleocapsid protein,	B Cell
246	*63856*	TGIEPDDHLKERSS	nucleocapsid protein,	B Cell
247	*64317*	TIGVYVIGFTLPII	nucleocapsid protein,	B Cell
248	*64644*	TKPTDPTGIE	N protein,nucleocapsid protein,	B Cell
249	*64645*	TKPTDPTGIEPDDH	nucleocapsid protein,	B Cell
250	*64782*	TLETKLGELKRQL	nucleoprotein,	B Cell
251	*65006*	TLPIILKALYMLST	nucleocapsid protein,	B Cell
252	*65021*	TLQARQQTVSALED	nucleocapsid protein,	B Cell
253	*65210*	TMKAEELTPGRFRT	nucleocapsid protein,	B Cell
254	*65881*	TQIQVRNIMSPVMG	nucleocapsid protein,	B Cell
255	*65938*	TQSMGIQLDQRIIL	nucleocapsid protein,	B Cell
256	*66194*	TSFEDINGIRRPKH	nucleocapsid protein,	B Cell
257	*66354*	TSPDNIDSPNAPWV	nucleocapsid protein,	B Cell
258	*67017*	TVGTAEEKLK	nucleocapsid protein,	B Cell

259	51616	QMISPVMSV	Nucleoprotein,hypothetical protein,nucleocapsid protein,	MHC Binding
260	59708	SMLSYGNVL	Nucleoprotein,hypothetical protein,nucleocapsid protein,	MHC Binding
261	60318	SPVMGVIGF	nucleocapsid protein,	MHC Binding
262	61191	SSLRYGNVL	nucleocapsid protein,	MHC Binding
263	64724	TLAQSLIDV	Nucleoprotein,hypothetical protein,nucleocapsid protein,	MHC Binding
264	52779	QVRNIMSPVMGVIGFSFFVKDW	N protein,	T Cell
265	54068	RHLYVSMPTAQSTMK	nucleocapsid protein,	T Cell
266	55184	RPKHLYVSM	nucleocapsid protein,	T Cell
267	57017	SATIFADI	nucleocapsid protein,	T Cell
268	59737	SMPTAQSTM	nucleocapsid protein,	T Cell
269	60320	SPVMGVIGFSFFVKD	nucleocapsid protein,	T Cell
270	61190	SSLRYGNV	nucleocapsid protein,	T Cell
271	65587	TPGRFRTI	nucleocapsid protein,	T Cell
272	66195	TSFEDINGIRRPKHL	nucleocapsid protein,	T Cell
273	164647	EEINGIRKPR	nucleocapsid protein,	B Cell
274	150982	FEDINGIRRP	nucleocapsid protein,	B Cell
275	956355	GKNIGQDRDPTGVEPGDHLKERSALSYGNTLDLNSLDID	nucleocapsid protein,	B Cell
276	165026	LKDAEKAVEVDPDDVNKSTLQSRRAAV	nucleocapsid protein,	B Cell
277	956424	PIAGSLSGNPVNRD	nucleocapsid protein,	B Cell
278	165338	THEQQLVTARQKLKDAEKAVEVDPDDVNKSTLQSRRAAV	nucleocapsid protein,	B Cell
279	67018	TVGTAEEKLK	N protein,nucleocapsid protein,	B Cell
280	67042	TVKENKGTRIRFKD	nucleocapsid protein,	B Cell
281	67972	VDHFHLGDDMDPEL	nucleocapsid protein,	B Cell
282	68034	VDPDDVNKNTLQAR	nucleocapsid protein,	B Cell
283	68042	VDPTGLEPDDHLK	nucleoprotein,	B Cell
284	68420	VFACAPDRCP	N protein,nucleocapsid protein,	B Cell
285	68800	VGTAEEKLKKKSSF	nucleocapsid protein,	B Cell
286	69199	VKDWSERIREFMEK	nucleocapsid protein,	B Cell
287	69289	VKPGTPAQEI	nucleocapsid protein,	B Cell
288	69441	VLDVNAIDIE	N protein,nucleocapsid protein,	B Cell
289	70109	VNKNTLQARQQTVSA	nucleocapsid protein,	B Cell
290	70879	VSALEDKLAD	N protein,nucleocapsid protein,	B Cell
291	70878	VSALEDKLAD	nucleocapsid protein,	B Cell
292	72183	VYVIGFTLPIILKA	nucleocapsid protein,	B Cell
293	73067	WSERIREFMEKECP	nucleocapsid protein,	B Cell
294	73191	WVFACAPDRCPPTC	nucleocapsid protein,	B Cel
295	73529	YDDVNGIRKP	nucleocapsid protein,	B Cell
296	151073	YEDVNGIRK	nucleocapsid protein,	B Cell
297	73645	YEEINGIRRP	nucleocapsid protein,	B Cell
298	74501	YKRRMADAVS	nucleocapsid protein,	B Cell
299	74503	YKRRMADAVSRKKM	nucleocapsid protein,	B Cell
300	76234	YVAGMAELGAFFSI	nucleocapsid protein,	B Cell
301	92496	DWMDRIEEF	nucleocapsid protein,	MHC Binding
302	92574	ELRQLAQSL	nucleocapsid protein,	MHC Binding
303	164233	FVVPILLKA	Nucleoprotein,hypothetical protein,nucleocapsid protein,	MHC Binding
304	92998	ILYMLSWGK	nucleocapsid protein,	MHC Binding
305	93361	LYVAGVPEL	nucleocapsid protein,	MHC Binding
306	93437	MRNTIMASK	nucleocapsid protein,	MHC Binding

307	*93759*	RQKLKDAEK	nucleocapsid protein,	MHC Binding
308	*93762*	RRAAVSTLE	nucleocapsid protein,	MHC Binding
309	*69961*	VMGVIGFGF	nucleocapsid protein,	MHC Binding
310	*72480*	WGKEAVNHF	nucleocapsid protein,	MHC Binding
311	*72661*	WKAIGAYIL	nucleocapsid protein,nucleoprotein,	MHC Binding
312	*72730*	WLIEPCKLL	nucleocapsid protein,	MHC Binding
313	*74297*	YILGFAIPI	nucleocapsid protein,nucleoprotein,	MHC Binding
314	*94311*	YLRQRQAAL	nucleocapsid protein,	MHC Binding
315	*74946*	YLTSFVVPI	nucleocapsid protein,	MHC Binding
316	*75086*	YMLSWGKEA	nucleocapsid protein,	MHC Binding
317	*94352*	YRTAVCGLY	Nucleoprotein,hypothetical protein,nucleocapsid protein,	MHC Binding
318	*130714*	AAECPFLPKPKVASE	nucleocapsid protein,nucleoprotein,	T Cell
319	*130779*	DWMDRIEEFLAAECP	nucleocapsid protein,nucleoprotein,	T Cell
320	*130796*	EEITPGRFRTIACGL	nucleocapsid protein,nucleoprotein,	T Cell
321	*149047*	FSILQDMRNTI	Nucleoprotein,hypothetical protein,nucleocapsid protein,	T Cell
322	*130849*	GFFVKDWMDRIEEFL	nucleocapsid protein,nucleoprotein,	T Cell
323	*130875*	GVIGFGFFVKDWMDR	nucleocapsid protein,nucleoprotein,	T Cell

324	*130892*	IEEFLAAECPFLPKP	nucleocapsid protein,nucleoprotein,	T Cell
325	*130897*	ILGFAIPIILKALYM	nucleocapsid protein,nucleoprotein,	T Cell
326	*130902*	IPIILKALY	nucleocapsid protein,nucleoprotein,	T Cell
327	*130903*	IPIILKALYMLSTRG	nucleocapsid protein,nucleoprotein,	T Cell
328	*130929*	KSTLQSRRAAVSTLE	nucleocapsid protein,nucleoprotein,	T Cell
329	*130932*	KVASEAFMSTNKMYF	nucleocapsid protein,nucleoprotein,	T Cell
330	*130948*	LKRQLADLVAAQKLA	nucleocapsid protein,nucleoprotein,	T Cell
331	*131049*	RQTVKDNKGTRIRFK	nucleocapsid protein,nucleoprotein,	T Cell
332	*131067*	SIDLEEPSGQTADWK	nucleocapsid protein,nucleoprotein,	T Cell
333	*131086*	SRRAAVSTLETKLGE	nucleocapsid protein,nucleoprotein,	T Cell
334	*131090*	STMKAEEITPGRFRT	nucleocapsid protein,nucleoprotein,	T Cell
335	*68421*	VFACAPDRCPPTCIYVA	N protein,	T Cell
336	*70789*	VRNIMSPVM		nucleocapsid protein,

Table S3. Pair of residues, which were mutated to cysteine amino acid.

TLR4-agonist vaccine	Chi3-angle	Energy (kcal/mol)	β-defensin vaccine	Chi angle	Energy (kcal/mol)	50S Ribosomal protein L7/L12 vaccine	Chi3-angle	Energy (kcal/mol)
Met19-Lys22	93.8	5.45	Cys18-Cys21	106.89	4.07	Met16-Ala69	-58.77	6.58
Gly28-Gly32	80.65	5.45	Ser22-Lys44	-113.3	7.26	Lys26-Glu33	85.73	1.18
Gly41-Asp46	90.89	4.71	Pro25-Gly37	111.43	2.85	Ala41-Ala45	-88.87	1.5
Asp46-Gly57	-91.92	2.94	Ser34-Gly37	-71.76	3.92	Glu55-Ser61	113.9	1.46
Ser52-Arg55	101.4	5.14	Asn54-Gly68	74.08	4.3	Val65-Ile78	105	1.95
Asp61-Ala64	102.95	1.6	Thr55-Gly66	-58.65	1.99	Leu67-Ile75	111.26	4.87
Thr74-Gly82	116.24	2.93	Thr61-Ala65	-58.42	7.28	Arg82-Gly89	115.16	1.78
Ala75-Lys86	74.54	5.57	Lys87-Tyr94	125.6	5.18	Val85-Leu88	118.05	2.84
Gly97-Gly101	-116.65	7.3	Asp99-Ala102	-75.9	6.36	Ala93-Ala100	-113.98	5.75
Arg110-Val113	119.28	3.07	Gly120-Lys124	121.86	4.57	Lys94-Pro101	122.86	3.7
Ala126-Ser202	114.88	3.18	Gln131-Gly135	67.41	6.6	Ala118-Ala125	-86.91	2.62
Pro132-Ser143	-88.91	1.1	Tyr184-Asn198	114.39	3.55	Met142-Ala150	109.15	3.91
Gly134-Glu140	101.26	5.19	Gly185-Ala202	-78.71	4.4	Pro154-Thr157	109.15	3.91
Gly138-Val149	-115.76	6.32	Pro193-Pro214	93.62	1.12	Pro154-Thr157	124.89	7.26
Gly147-Thr167	-87.26	1.09	Thr199-Lys204	71.69	3.24	Lys179-Tyr1179	96.31	5.2
Val152-Val168	123.82	5.83	Ala209-Lys216	104.4	3.45	Ser175-Arg178	117.23	3.82
Asn160-Gly169	126.76	3.22	Gly215-Ile218	117.21	4.64	Ile188-Gly201	-60.14	2.08
Glu172-Gly175	89.27	4.4	Asp231-Thr244	85.94	2.59	Glu192-Thr197	-84.76	2.42
Gly173-Pro186	114.88	4.14	Glu235-Ala238	115.05	4.73	Gly195-Gly203	112.31	3.41
Asp208-Ser211	103.01	2.54	Tyr241-Asn243	122.58	3.46	Pro202-Val210	110.58	4.31
Ile214-Pro219	114.24	2.42	Leu264-Leu270	121.98	5.25	Ile206-Lys209	123.64	4.45
Gly220-Pro237	-58.29	3.29	Asp279-Glu282	98.01	5.57	Gly240-Thr290	74.96	5.78
Glu227-Asp230	-101.5	3.39	Thr287-Lys291	104.83	5.9	Gly270-Val291	108.97	6.21
Gly238-Pro257	101.6	4.68	-	-	-	Asp306-Gly311	-97.35	1.55
Ser239-Pro255	101.84	3.2	-	-	-	Leu330-Asn333	75.42	6.15
Ser246-Gly254	-91.74	2.57	-	-	-	Ser362-Leu365	123.02	3.16
